# Mechanism of imidazole inhibition of a GH1 β‐glucosidase

**DOI:** 10.1002/2211-5463.13595

**Published:** 2023-03-25

**Authors:** Rafael S. Chagas, Felipe A. M. Otsuka, Mario A. R. Pineda, Roberto K. Salinas, Sandro R. Marana

**Affiliations:** ^1^ Departamento de Bioquímica, Instituto de Química Universidade de São Paulo Brazil

**Keywords:** enzyme kinetics, GH1 β‐glucosidases, imidazole, inhibition mechanism, partial competitive, *Spodoptera frugiperda*

## Abstract

Imidazole is largely employed in recombinant protein purification, including GH1 β‐glucosidases, but its effect on the enzyme activity is rarely taken into consideration. Computational docking suggested that imidazole interacts with residues forming the active site of the GH1 β‐glucosidase from *Spodoptera frugiperda* (Sfβgly). We confirmed this interaction by showing that imidazole reduces the activity of Sfβgly, which does not result from enzyme covalent modification or promotion of transglycosylation reactions. Instead, this inhibition occurs through a partial competitive mechanism. Imidazole binds to the Sfβgly active site, reducing the substrate affinity by about threefold, whereas the rate constant of product formation remains unchanged. The binding of imidazole within the active site was further confirmed by enzyme kinetic experiments in which imidazole and cellobiose competed to inhibit the hydrolysis of *p*‐nitrophenyl β‐glucoside. Finally, imidazole interaction in the active site was also demonstrated by showing that it hinders access of carbodiimide to the Sfβgly catalytic residues, protecting them from chemical inactivation. In conclusion, imidazole binds in the Sfβgly active site, generating a partial competitive inhibition. Considering that GH1 β‐glucosidases share conserved active sites, this inhibition phenomenon is probably widespread among these enzymes, and this should be taken into account when considering the characterization of their recombinant forms.

## Graphical abstract

Imidazole is largely employed in recombinant protein purification, including of GH1 β‐glucosidases, but its effect on the enzyme activity is overlooked. Computational docking suggested that imidazole interacts within the active site of a GH1 β‐glucosidase. By using enzyme kinetics experiments, we demonstrated that this inhibition occurs through a partial competitive mechanism, reducing the substrate affinity by about threefold.

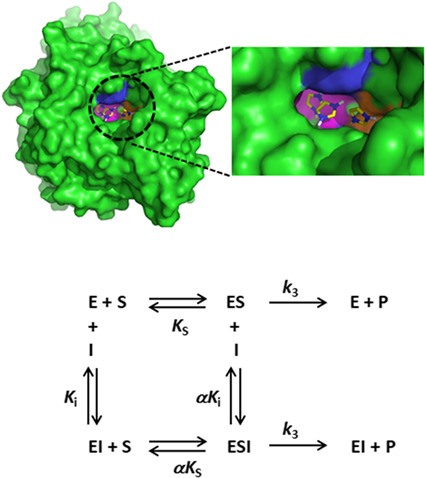

**Abbreviations**
EDC: 1‐ethyl‐3‐(dimethylamino‐propyl) carbodiimide
GEE: glycine ethyl ester
GH: glycoside hydrolases
Sfβgly: GH1 β‐glucosidase from *Spodoptera frugiperda*

Glycoside hydrolases (GH), based on their sequence and structural similarities, are grouped into more than 170 families in the CAZY databank (http://www.cazy.org) [1, 2]. Among these families, the GH1 family comprehends β‐glycosidases, enzymes that hydrolyze the O‐ or S‐glycosidic linkages of β‐glycosides, and fold as TIM barrels ([β/α]_8_ barrel) [3], a repetition of a (β/α) motif [4]. β‐glucosidases enzymes (3.2.1.21) are overspread among all forms of life, from bacteria to eukaryotes [5], and are related to a series of biological functions such as digestion, defense, and plant cell wall metabolism [6, 7, 8].

β‐glucosidases catalyze glycoside hydrolysis via a double‐displacement mechanism involving two carboxylic acid‐containing side chains in the active site. One of these groups, a carboxylate, functions as a nucleophile, leading to a glucosyl–enzyme intermediate. The other carboxylate acts as a general acid catalyst in the formation of this intermediate, and as a general base catalyst in its breakdown promoted by water [9]. Inhibitors of these enzymes, responsible for the disruption of their activity, have played a vital role in revealing their functions in the living system by modifying or blocking specific metabolic processes [10], leading to several applications of these chemicals in agriculture and medicine [11]. For instance, the finding that β‐glucosidases are linked to Gaucher's syndrome [12] prompted detailed investigations on compounds able to inhibit these enzymes. Therapeutic use of these inhibitors can be found in the control of this disease, which is related to disturbed lysosomal storage [12]. Besides the therapeutic applications, β‐glucosidase inhibitors are also useful for probing the binding properties of these enzymes and may also be used as a tool for investigating and elucidating their mechanism of catalysis [13, 14, 15, 16].

Here, we investigated the effect of imidazole, a largely employed reagent in the recombinant protein purification [17, 18], as an inhibitor of the recombinant GH1 β‐glucosidase from the fall armyworm *Spodoptera frugiperda* (hereafter called Sfβgly). Sfβgly is a secreted digestive enzyme that is associated with the glycocalyx of midgut epithelial cells [6], and it was previously studied regarding substrate recognition, catalysis, thermal stability, and oligomerization [6, 19, 20, 21, 22]. The Sfβgly crystallographic structure (PDB ID: 5CG0) [20] shows an active site divided into subsites, among which the subsite −1, which binds the monosaccharide of the substrate nonreducing end, is shaped by a set of residues that form hydrogen bonds with the ligand [23], substrate glycones, or inhibitors, which indicate a potential interaction site with imidazole. Imidazole derivatives have been shown before as a β‐glucosidase inhibitor [13, 14, 15, 16, 24]; however, those experiments were conducted with nonhomogeneous sweet almond β‐glucosidase samples. In addition, only low concentrations of the derivatives were tested [16]. Here we performed experiments with a purified GH1 β‐glucosidase (Sfβgly), which crystallographic structure is already known. In addition, we used higher concentrations of imidazole, the same reagent and conditions employed in the recombinant protein purification. That led to the characterization of the mechanism of inhibition of this GH1 β‐glucosidase by imidazole. Finally, considering the conservation of the residues forming their active sites, this inhibition mechanism is probably widespread among GH1 β‐glucosidases, information that should be taken into account in the characterization of these recombinant enzymes, reinforcing the relevance of the imidazole removal after the protein purification steps.

## Results and Discussion

**Fig. 1:**
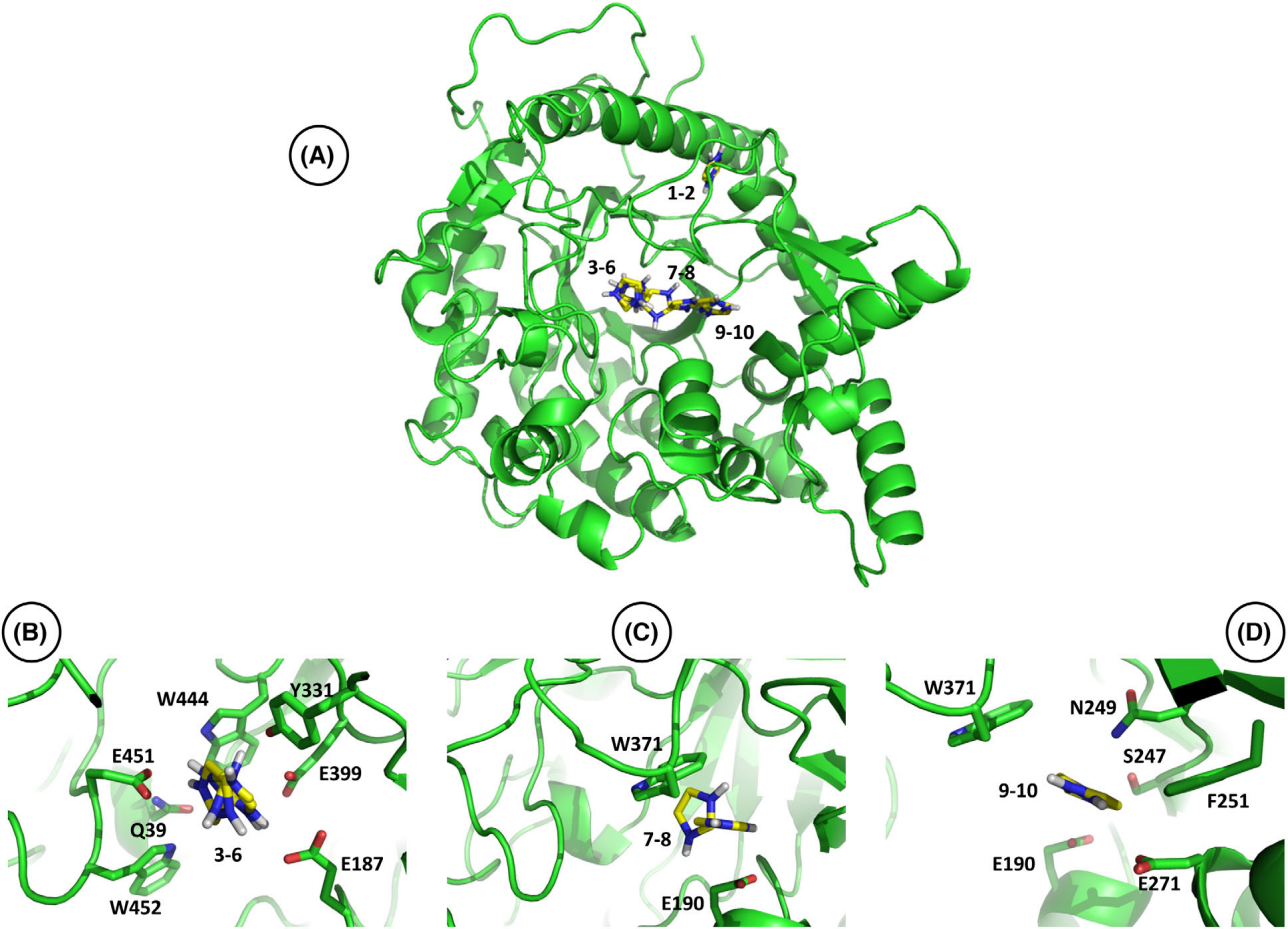
Structural mapping of the docking solutions for imidazole binding in the GH1 β‐glucosidase Sfβgly. (A) Superposition of the 10 best docking solutions. Solutions are numbered as presented in Table 1. (B) Different orientations of the imidazole within the Sfβgly active site (subsite −1) observed in the docking solutions 3–6. (C) Imidazole binding in the Sfβgly active site (subsite +1) observed in the docking solutions 7 and 8. (D) Imidazole interaction in the pocket close to the active site opening presented in the docking solutions 9 and 10. Residues Q39, E187, E190, S247, N249, F251, E271, Y331, W371, E399, W444, E451, and W452 are at 3.5 Å of the imidazole N_1_ or N_3_.

The potential interaction between imidazole and β‐glucosidase Sfβgly was initially evaluated by computational docking. The 10 best interaction solutions identified in the Autodock search included eight different orientations of the imidazole ring within the −1 and +1 subsites of the active site (solutions 3–10), and finally, two binding spots between residues S378 and S424 (solutions 1 and 2), which are part of a contact network that indirectly affects the Sfβgly substrate specificity [19] (Table 1; Fig. 1A). The structural analysis of the solutions 3–10, within the active site, showed residues Q39, E187, E190, S247, N249, F251, E271, Y331, W371, E399, W444, E451, and W452 at 3.5 Å from N_1_ or N_3_ of the imidazole ring, suggesting that they may form noncovalent interactions (Table 1). Residues E187 and E399 are the Sfβgly catalytic residues, Q39 and E451 form hydrogen bonds with the glycone OH4 of the substrate and W444 is the basal platform of the −1 subsite, which also forms contacts with the substrate glycone [6] (Fig. 1B), whereas residues E190 and W371 are part of the subsite +1 that interacts with the substrate aglycone [25] (Fig. 1C). Finally, residues S247, N249, F251, and E271, seen in the solutions 9 and 10, form a pocket very close to the subsite +1 within the active site opening (Fig. 1D). These residues are also central in the Sfβgly noncovalent interaction network [21].

**Table 1:** Docking solutions for imidazole binding in the GH1 β‐glucosidase Sfβgly.

Autodock solution	Binding score	Sfβgly residue within 3.5 Å of the imidazole nitrogen atoms	Sfβgly region
1	−4.0	S378, I379, and S424	Surface
2	−3.8	S378, I379, and S424	Surface
3	−3.6	Q39, E187, E451, and W452	Active site; −1 subsite
4	−3.6	E187, Y331, W371, E399, and E451	Active site; −1 and +1 subsites
5	−3.6	E187, W371, E399, W444, and E451	Active site; −1 and +1 subsites
6	−3.4	E187, Y331, W371, E399, W444, and E451	Active site; −1 and +1 subsites
7	−3.2	E187, E190, S247, and N249	Active site; +1 subsite and pocket
8	−3.1	W143, E187, E190, and Y331	Active site; −1 and +1 subsite
9	−3.0	R189, E190, S247, N249, and F251	Active site; +1 subsite and pocket
10	−3.0	R189, E190, S247, N249, F251, and E271	Active site; +1 subsite and pocket

In short, the docking solutions confirm the imidazole interaction within and close to the β‐glucosidase Sfβgly active site is a reasonable possibility, which then could have effects on the enzyme activity.

**Fig. 2:**
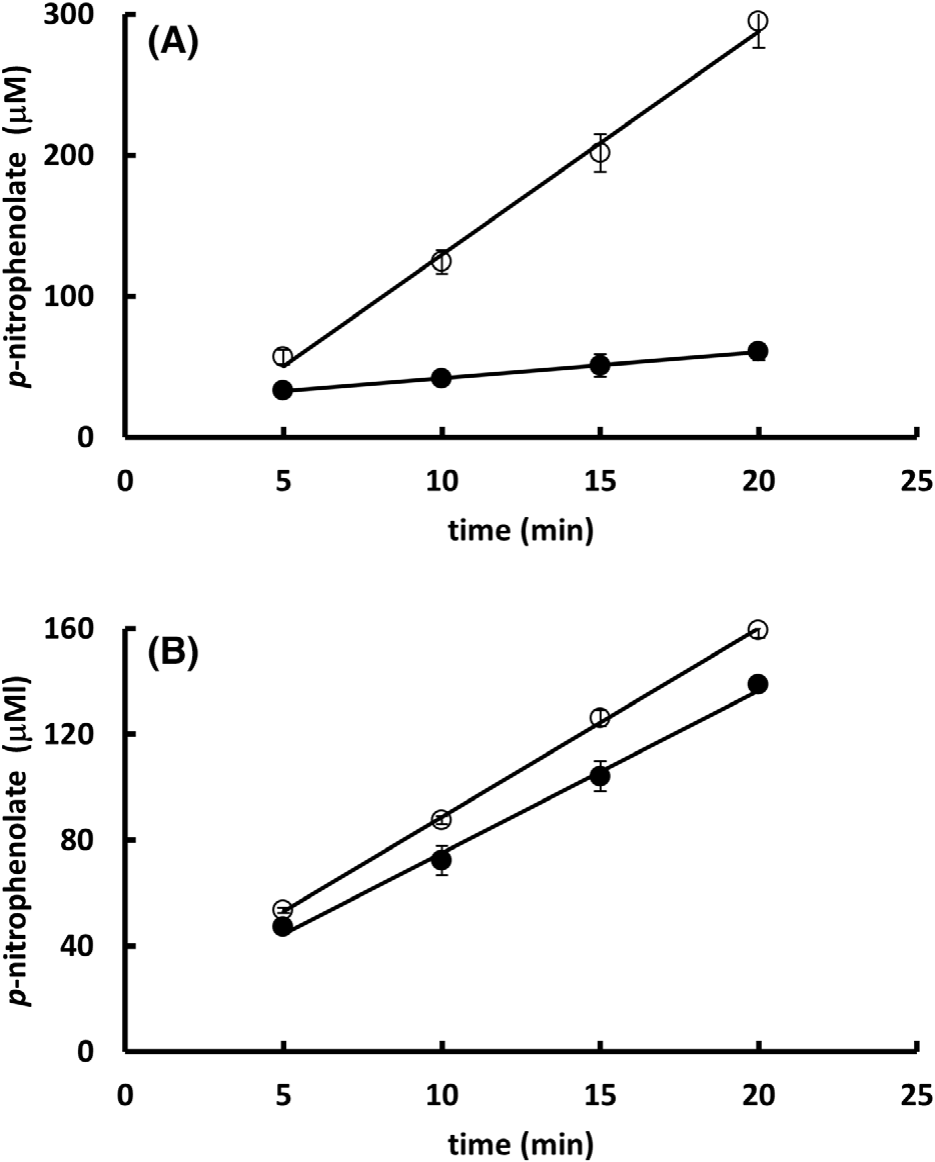
Effect of imidazole on the activity of the β‐glucosidase Sfβgly. (A) Determination of the initial rate of the 15 mm *p*‐nitrophenyl β‐glucoside hydrolysis catalyzed by Sfβgly in the absence (○) and presence (●) of 150 mm imidazole. Initial rates are 16 and 2 μm·min^−1^ in the absence and presence imidazole, respectively. Sfβgly concentration was 0.1 μm. (B) Determination of the initial rate of the 15 mm *p*‐nitrophenyl β‐glucoside hydrolysis catalyzed by Sfβgly previously incubated at 30 °C for 18 h with 150 mm imidazole (●) and without imidazole (○). Rates are 6 and 7 μm·min^−1^, respectively. Sfβgly concentration was 0.05 μm. Data are mean and standard deviation of three determinations of the product formed in each incubation time using three separate assays with the same enzyme sample. The substrate was prepared in 100 mm phosphate buffer pH 6.0. Activity assays were performed at 30 °C.

Indeed, we observed that 150 mm imidazole, when present in the reaction mix, reduced the initial rate of substrate hydrolysis catalyzed by the Sfβgly to 12% (Fig. 2A). That inhibitory effect does not arise from enzyme inactivation since the previous incubation of Sfβgly at 30 °C with 150 mm imidazole for 18 h followed by an activity assay in ‘imidazole‐free’ conditions did not significantly change the Sfβgly activity (Fig. 2B).

**Fig. 3:**
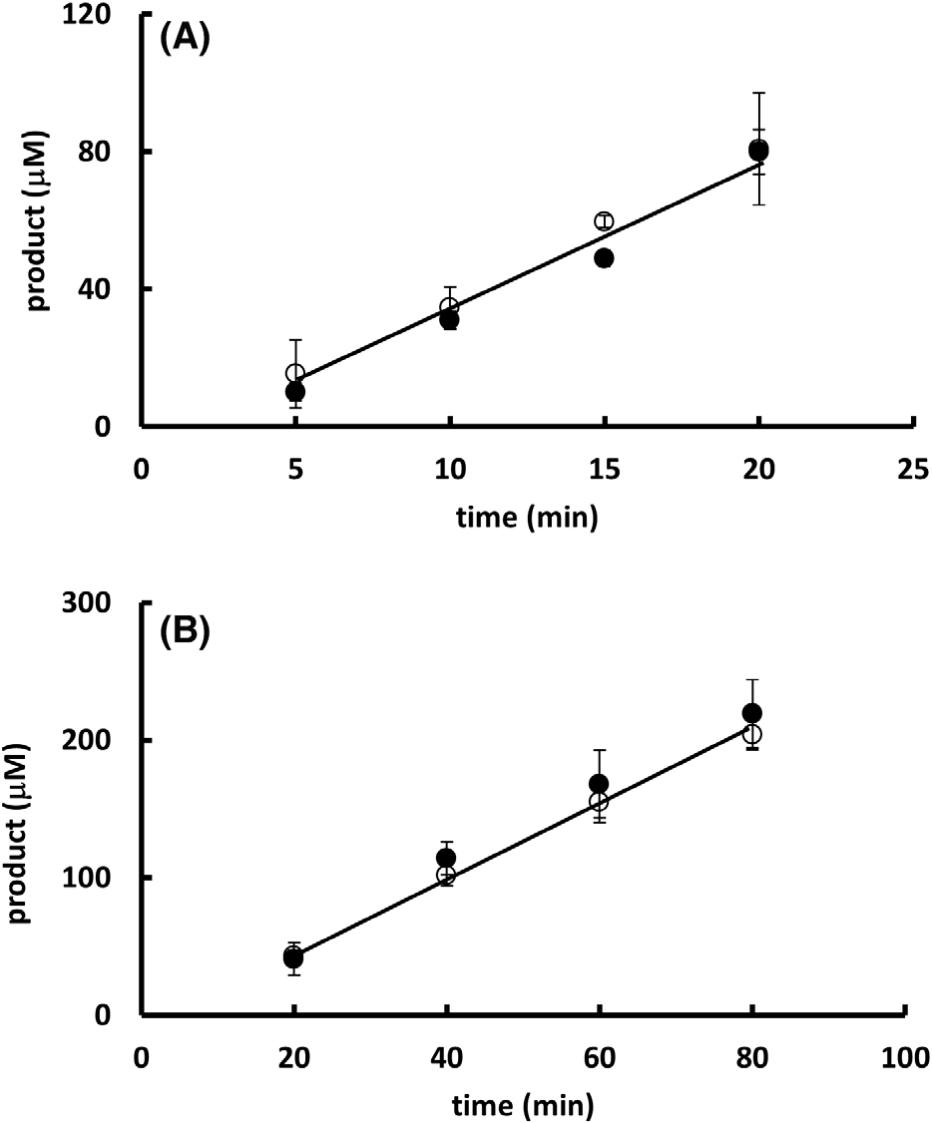
Evaluation of transglycosylation reactions catalyzed by the β‐glucosidase Sfβgly. (A) Determination of the activity of the Sfβgly followed by the production of *p*‐nitrophenolate (○) and glucose (●). (B) Determination of the activity of the Sfβgly followed by the production of *p*‐nitrophenolate (○) and glucose (●) in the presence of 150 mm imidazole. The substrate was 15 mm *p*‐nitrophenyl β‐glucoside prepared in 100 mm phosphate buffer pH 6.0. Sfβgly concentration was 0.05 μm. Data are mean and standard deviation of three determinations of the product formed in each incubation time using the same enzyme sample. Activity assays were performed at 30 °C.

The possibility that the inhibitory effect actually resulted from the participation of imidazole as an acceptor in transglycosylation reactions catalyzed by Sfβgly was evaluated by following the formation of the products glucose and *p*‐nitrophenolate from the substrate *p*‐nitrophenyl β‐glucoside. It was observed that those products are formed in a 1 : 1 ratio (4.4 and 4.5 μ m·min^−1^; Fig. 3) in reactions performed without imidazole. This ratio also holds even when 150 mm imidazole was present (2.7 and 2.9 μ m·min^−1^; Fig. 3). Considering that the occurrence of transglycosylation reaction should reduce the rate of glucose production, which would be incorporated in the transglycosylation product, the observed 1 : 1 ratio indicates the absence of transglycosylation reactions in both conditions (Fig. 3).

**Fig. 4:**
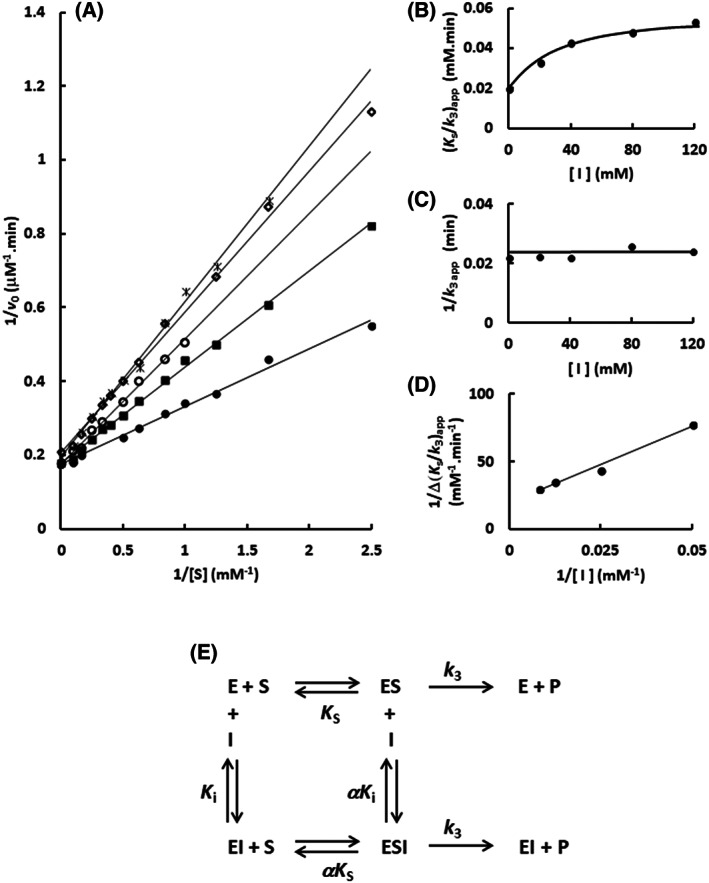
Determination of the mechanism of Sfβgly inhibition by imidazole using the substrate *p*‐nitrophenyl β‐glucoside. (A) Lineweaver–Burk plots representing the effect of the *p*‐nitrophenyl β‐glucoside concentration on the initial rate of substrate hydrolysis in the presence of different imidazole concentrations: (●) 0; (■) 20 mm; (○) 40 mm; (◊) 80 mm; (*) 120 mm. (B) Effect of the imidazole concentration on the apparent *K*_S_/*k*_3_ (calculated from the line slope in the Lineweaver–Burk plot). (C) Effect of the imidazole concentration on the 1/*k*_3_ apparent (calculated from the line intercept in the Lineweaver–Burk plot). (D) Secondary plot (1/Δ_*K*s/*k*3 app_ *versus* 1/[I]) representing the effect of the imidazole concentration on the apparent *K*_S_/*k*_3_. Δ_*K*s/*k*3 app_ = *K*_S_/*k*_3i_ – *K*_S_/*k*_30_, in which *K*_S_/*k*_3i_ corresponds to the line in the presence of imidazole and *K*_S_/*k*_30_ to the line in the absence of imidazole. The substrate and inhibitor were prepared in 100 mm phosphate buffer pH 6.0. Activity assays were performed at 30 °C. The results presented above correspond to the average of experiments repeated in the same conditions with three different enzyme samples (0.125 μm; Figs [Link], [Link]). (E) Schematic representation of the partial competitive inhibition mechanism observed for imidazole. I corresponds to imidazole; *S* represents the substrate. E is the GH1 β‐glucosidase Sfβgly. P is the product. *K*_s_ is the dissociation constant for the enzyme–substrate (ES) complex. *K*_i_ is the dissociation constant for the enzyme–inhibitor (EI) complex. *k*_3_ is the rate constant for the product formation from the ES complex. The α factor represents the hindering effect between the substrate and inhibitor (α > 1), based on [26].

Hence, an enzyme kinetics approach was used to determine the mechanism of imidazole inhibition upon Sfβgly. The initial rate of substrate hydrolysis was determined in different *p*‐nitrophenyl β‐glucoside concentrations in the presence of several fixed imidazole concentrations. Lineweaver–Burk plots were used to analyze the data revealing a hyperbolic relation between the apparent *K*_s_/*k*_3_ (calculated from the line slope) and imidazole concentration, whereas the apparent 1/*k*_3_ (calculated from the line intercept) remained constant (Fig. 4A–D). Those are features of a partial competitive inhibition mechanism (simple intersecting hyperbolic competitive; [26]), which is depicted in Fig. 4E.

In this partial competitive inhibition mechanism, the constant line intercept in the 1/*v*_0_ × 1/[*S*] plot (which is proportional to 1/*k*_*3*_) indicates that the *p*‐nitrophenyl β‐glucoside and imidazole may bind simultaneously in the enzyme forming a ternary complex ESI that is as productive as the ES complex, that is, exhibits the same *k*_3_. However, the imidazole interaction with the enzyme reduces the affinity for the substrate, that is, increases the *K*_S_ by a factor termed α (α > 1). The reverse is also true. The substrate binding reduces the affinity between the enzyme and imidazole, that is, also increases the *K*_i_ by the same α factor. Moreover, as the imidazole concentration increases, the complete enzyme population is driven to the EI and the ESI complexes, which bring about the same *V*_max_ but a higher *K*_s_. Therefore, the imidazole inhibitory effect results from the apparent lower substrate affinity. Thus, as the enzyme population shifts from E and ES to the EI and ESI states, the slope (which is proportional to *K*_s_/*k*_3_) of the lines in the Lineweaver–Burk plot increases and reaches a maximum (α*K*_s_/*k*_3_), producing the already mentioned hyperbolic curve in the *K*_s_/*k*_3 app_ *vs* [imidazole] plot, whereas the *V*_max_ remains constant as shown in the 1/*k*_3 app_ *vs* [imidazole] plot (Fig. 4). Based on the expression describing the line slope in the Lineweaver–Burk plot [26], a secondary plot (1/Δ_*K*s/*k*3 app_ *vs* 1/[imidazole]) was produced, from which the *K*_i_ for imidazole, 15.3 ± 0.3 mm, and its effect on the *K*_s_, α = 3 ± 1, were evaluated (Fig. 4; Figs [Link], [Link]).

**Fig. 5:**
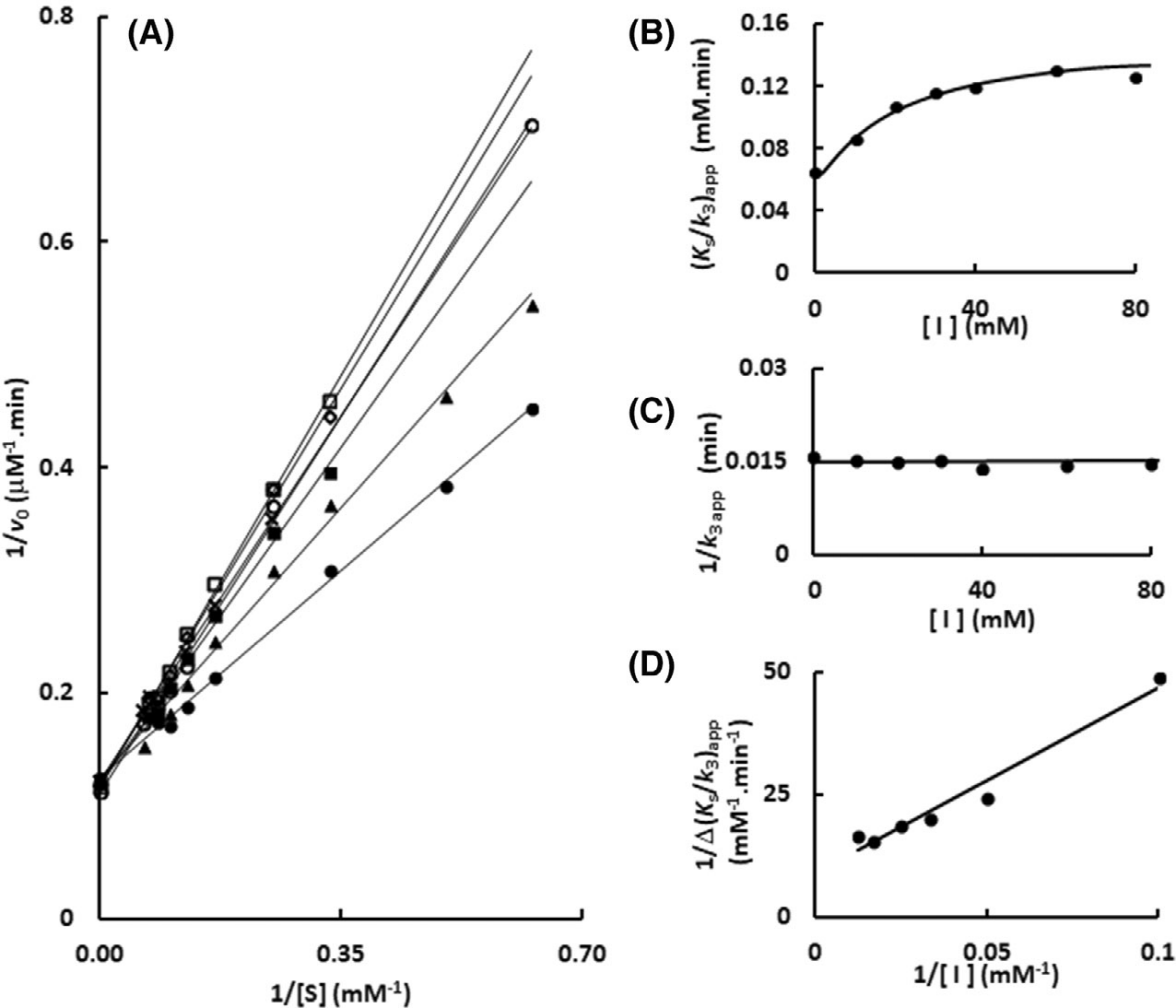
Determination of the mechanism of Sfβgly inhibition by imidazole using the substrate cellobiose. (A) Lineweaver–Burk plots representing the effect of the cellobiose concentration on the initial rate of substrate hydrolysis in the presence of different imidazole concentrations: (●) 0; (▲) 10 mm; (■) 20 mm; (×) 30 mm; (○) 40 mm; (□) 60 mm; (◊) 80 mm. (B) Effect of the imidazole concentration on the apparent *K*_S_/*k*_3_ (calculated from the line slope in the Lineweaver–Burk plot). (C) Effect of the imidazole concentration on the apparent 1/*k*_3_ (calculated from the line intercept in the Lineweaver–Burk plot). (D) Secondary plot (1/Δ_*K*S/*k*3 app_ *versus* 1/[I]) representing the effect of the imidazole concentration on the apparent *K*_S_/*k*_3_. Δ_*K*S/*k*3_ = *K*_S_/*k*_3i_ – *K*_S_/*k*_30_, in which *K*_S_/*k*_3i_ corresponds to the line in the presence of imidazole and *K*_S_/*k*_30_ to the line in the absence of imidazole. The substrate and inhibitor were prepared in 100 mm phosphate buffer pH 6.0. Activity assays were performed at 30 °C. The results presented above correspond to the average of experiments repeated in the same conditions with three different enzyme samples (0.125 μm; Figs [Link], [Link] and S7).

**Fig. 6:**
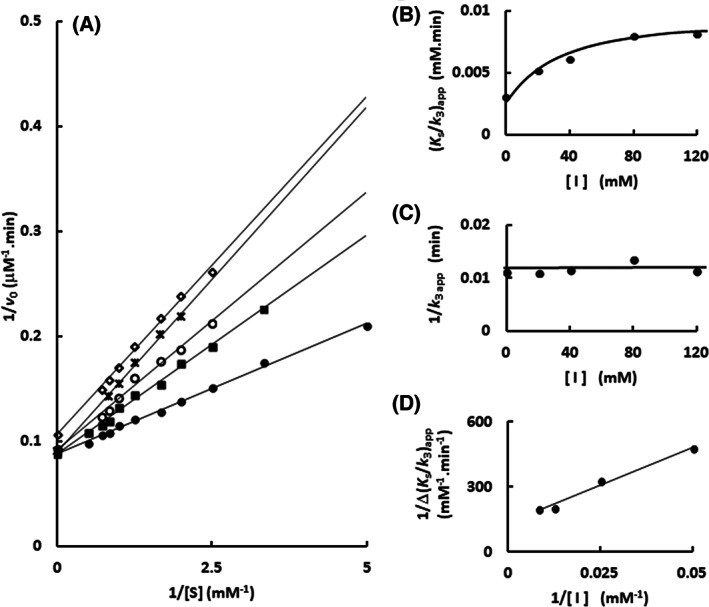
Determination of the mechanism of Sfβgly inhibition by imidazole using the substrate cellotetraose. (A) Lineweaver–Burk plots representing the effect of the cellotetraose concentration on the initial rate of substrate hydrolysis in the presence of different imidazole concentrations: (●) 0; (■) 20 mm; (○) 40 mm; (◊) 80 mm; (×) 120 mm. (B) Effect of the imidazole concentration on the apparent *K*_S_/*k*_3_ (calculated from the line slope in the Lineweaver–Burk plot). (C) Effect of the imidazole concentration on the apparent 1/*k*_3_ (calculated from the line intercept in the Lineweaver–Burk plot). (D) Secondary plot (1/Δ_*K*S/*k*3 app_ *versus* 1/[*I*]) representing the effect of the imidazole concentration on the apparent *K*_S_/*k*_3_. Δ_*K*S/*k*3 app_ = *K*_S_/*k*_3i_ – *K*_S_/*k*_30_, where *K*_S_/*k*_3i_ corresponds to the line in the presence of imidazole and *K*_S_/*k*_30_ to the line in the absence of imidazole. The substrate and inhibitor were prepared in 100 mm phosphate buffer pH 6.0. Activity assays were performed at 30 °C. The results presented above correspond to the average of experiments repeated in the same conditions with three different enzyme samples (0.125 μm; Figs [Link], [Link] and S10).

The same approach was used to characterize the imidazole inhibitory mechanism upon different substrates. These substrates range from cellobiose (C2; Fig. 5; Figs [Link], [Link]), which occupies the subsites −1 and +1 similarly to the *p*‐nitrophenyl β‐glucoside, to a larger oligocellodextrin, cellotetraose (C4; Fig. 6; Figs [Link], [Link]), which fill subsites beyond +1, even reaching the active site opening. For comparative purpose, the kinetic parameters for hydrolysis of those substrates are reported in Table S1.

The same imidazole inhibitory mechanism, *K*_i_ and α were observed for these oligocellodextrins (Table 2; Figs 4, 5, 6; Figs [Link], [Link]). Indeed, the extension of the substrate, that is, the subsites filled, did not change the mutual hindrance between imidazole and substrate. Therefore, imidazole binds at the same region of the Sfβgly active site in the presence of these different substrates.

**Table 2:** Parameters of the imidazole inhibitory mechanism upon the hydrolysis of different substrates by the Sfβgly. The inhibition mechanism is partial competitive as depicted in the Fig. 4E. The α factor represents the mutual hindrance between imidazole and substrate. *K*_i_ is the dissociation constant for the enzyme–imidazole complex. Data are mean and standard deviation based on three independent experiments (Figs [Link], [Link]). NPβglc, *p*‐nitrophenyl β‐glucoside; C2, cellobiose; C4, cellotetraose. Kinetic parameters for the substrate hydrolysis are reported in the Table S1.

Substrate	*K*_i_ (mm)	α
NPβglc	15.3 ± 0.3	3 ± 1
C2	14 ± 3	2.3 ± 0.1
C4	15 ± 5	2.8 ± 0.7

**Fig. 7:**
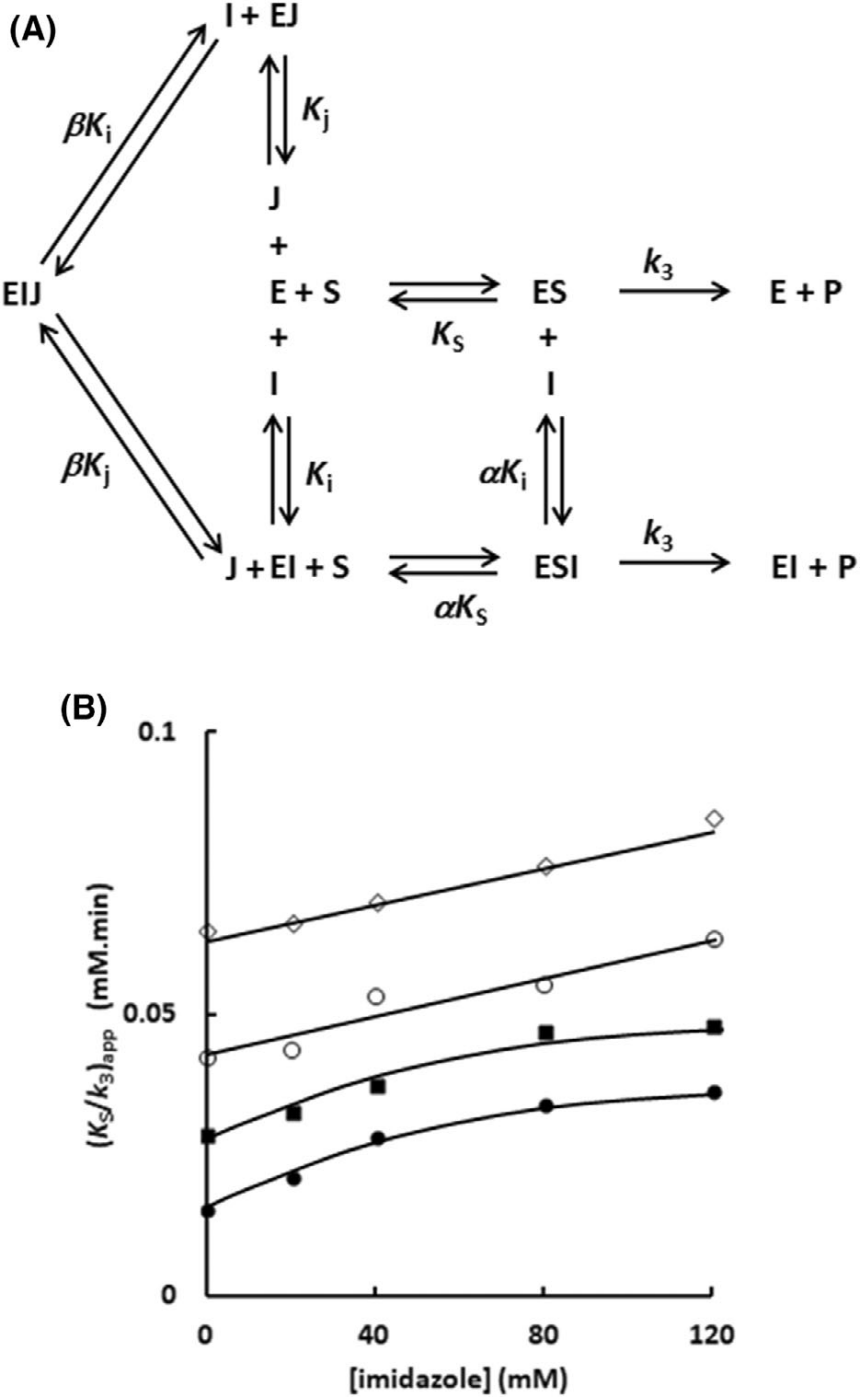
Simultaneous effect of imidazole and cellobiose on the Sfβgly hydrolysis of the substrate. (A) Schematic representation of the simultaneous effect of imidazole (*I*; a partial competitive inhibitor) and cellobiose (*J*; a competitive inhibitor) on the substrate (*S*; *p*‐nitrophenyl β‐glucoside) hydrolysis. E, GH1 β‐glucosidase Sfβgly; P—product. *K*_s_ is the dissociation constant for the enzyme–substrate complex (ES). *K*_i_ is the dissociation constant for the enzyme–imidazole complex (EI). *K*_j_ is the dissociation constant for the enzyme–cellobiose complex (EJ). The α factor represents the hindering effect between the substrate and imidazole (Table 2). The β factor represents the hindering effect between imidazole and cellobiose. (B) Effect of the imidazole concentration on the apparent *K*_s_/*k*_3_ (calculated from the line slope in the Lineweaver–Burk plot) in the presence of constant cellobiose concentration. The initial rates of the hydrolysis of several *p*‐nitrophenyl β‐glucoside concentrations were determined in the presence of different imidazole concentrations. The constant cellobiose concentrations were (●) 5, (■) 10, (○) 20, and (◊) 30 mm, which correspond to 0.5, 1, 2, and 3 *K*_i_. Lineweaver–Burk plots resulting from these experiments are presented in Figs [Link], [Link]. The substrate and inhibitors were prepared in 100 mm phosphate buffer pH 6.0. Activity assays were performed at 30 °C.

Next, the interaction of the imidazole with the Sfβgly was investigated using a different approach, which is depicted in Fig. 7. The enzyme and substrate were simultaneously combined with imidazole and a competitive inhibitor. Assuming that both bind in the active site, the presence of the competitive inhibitor should hinder the imidazole binding, hence altering its inhibitory effect upon the substrate hydrolysis. On the contrary, if imidazole does not bind within the active site, the presence of a competitive inhibitor would not influence the imidazole inhibitory effect. Hence, in this approach, we seek to determine the effect that cellobiose, as a competitive inhibitor, exerts upon the imidazole inhibitory effect.

Cellobiose was used as this second competitive inhibitor because, as a natural substrate, it would surely bind in the Sfβgly active site. Moreover, when the *p*‐nitrophenyl β‐glucoside is the substrate and the initial rate of reaction is determined by following the formation of *p*‐nitrophenolate, cellobiose behaves as a competitive inhibitor. Indeed, that was demonstrated and the *K*_i_ for cellobiose is 10 mm (Fig. S11). Finally, it should be noted that the *k*_3_/*K*_S_ for the cellobiose hydrolysis is about 3 times lower than the *p*‐nitrophenyl β‐glucoside (Table S1). Hence, the initial rate condition applies to both, indicating that cellobiose concentration is also approximately constant during the experiment time.

The initial rates of the hydrolysis of several *p*‐nitrophenyl β‐glucoside concentrations were determined in the presence of increasing fixed imidazole concentration, similarly to the experiments reported above (Fig. 4). However, now these experiments were performed in the presence of constant cellobiose concentration, 5, 10, 20 and 30 mm that correspond to 0.5, 1, 2, and 3 *K*_i_ (Figs [Link], [Link]). So, these cellobiose concentrations should produce a gradient of increasing concentration of the Sfβgly–cellobiose complex, that is, the EJ complex in Fig. 7A, diverting the enzyme away from the complexes with imidazole (EI and ESI). Again, we analyzed the imidazole inhibitory effect by following the changes in the apparent *K*_s_/*k*_3_ calculated from the line slopes in the Lineweaver–Burk plots (Fig. 7B and Figs [Link], [Link]).

At 5 mm cellobiose (0.5 *K*_i_), the imidazole inhibitory effect is observed again as the hyperbolic behavior of the apparent *K*_s_/*k*_3_ *vs* [imidazole] plot. However, at 30 mm cellobiose (3*K*_i_) the apparent *K*_s_/*k*_3_ increases linearly with imidazole concentration (Fig. 7B). Hence the increment of the cellobiose concentration is shifting the inhibitory effect away from the partial competitive mechanism. Indeed, that is expected if both inhibitors, cellobiose and imidazole, were competing for the same spot in the active site. The increment of cellobiose concentration would saturate the enzyme, hampering the imidazole binding (Fig. 7A). Moreover, in a saturating cellobiose concentration, the lines in the Lineweaver–Burk plot (Figs [Link], [Link]) would tend to the same apparent *K*_s_/*k*_3_, that is, slope, because the dominant inhibitory effect would result from the same cellobiose concentration. In agreement, the ratio between the apparent *K*_s_/*k*_3_ in the absence and in the presence of the higher imidazole concentration is about 2.5 at 5 mm cellobiose, but it decreases to 1.3 at 30 mm cellobiose (Fig. 7B). In short, the presence of cellobiose is abolishing the imidazole inhibitory effect.

Next, we moved to quantify the mutual hindering between cellobiose and imidazole when both act as inhibitors in the experiment reported above (Fig. 7; Figs [Link], [Link]). The value of such parameter, which was termed β in Fig. 7A, should be comparable to α, previously evaluated when using the imidazole as an inhibitor of the substrate hydrolysis (Table 2). Based on the scheme describing the simultaneous interaction of imidazole and cellobiose with Sfβgly (Fig. 7A), we deduced the rate equation below (Fig. S16) describing the lines in the Lineweaver–Burk plot presented in Figs [Link], [Link].

(1) $$\frac{1}{v_{o}}=\frac{K_{s}}{V_{\max}}\;\frac{\left(\alpha K_{i}+\alpha \left[I\right]+\frac{\alpha K_{i}\left[J\right]}{K_{j}}+\frac{\alpha \left[I\right]\left[J\right]}{\beta K_{j}}\right)}{\alpha K_{i}+\left[I\right]}\frac{1}{\left[S\right]}+1/V_{\max}.$$

*J* is cellobiose, *I* is imidazole, α represents the mutual impediment between *S* and imidazole (Table 2) and β corresponds to the mutual impediment between cellobiose and imidazole. The substrate *S* is *p*‐nitrophenyl β‐glucoside. *K*_s_ is the dissociation constant for the enzyme–substrate complex. *K*_i_ is the dissociation constant for the enzyme–imidazole complex (Table 2). *K*_j_ is the dissociation constant for the enzyme–cellobiose complex (Fig. S11).

As seen, the slope of this linear equation (i.e., apparent *K*_s_/*V*_max_) describes both inhibitor effects on the enzyme, whereas the intercept (1/*V*_max_) does not depend on their concentration. Hence, the slope was isolated and expressed in two limiting situations: infinite concentration of imidazole (slope_∞_) and absence of imidazole (slope_0_), both in the presence of the same cellobiose concentration.

(2) $${\text{slope}}_{\infty }=\frac{K_{s}}{V_{\max}}\;\alpha \;\left(1+\frac{\left[J\right]}{\beta K_{j}}\right)$$

(3) $${\text{slope}}_{0}=\frac{K_{s}}{V_{\max}}\;\left(1+\frac{\left[J\right]}{K_{j}}\right)$$

The ratio slope_∞_/slope_0_ results in the expression below, in which the β describes the mutual impediment between cellobiose and imidazole (*J* and *I*, respectively; Fig. 7A).

(4) $$\frac{{\text{slope}}_{\infty }}{{\text{slope}}_{0}}=\frac{\alpha }{\beta }\frac{\left(\beta K_{j}+\left[J\right]\right)}{\left(K_{j}+\left[J\right]\right)}$$

**Fig. 8:**
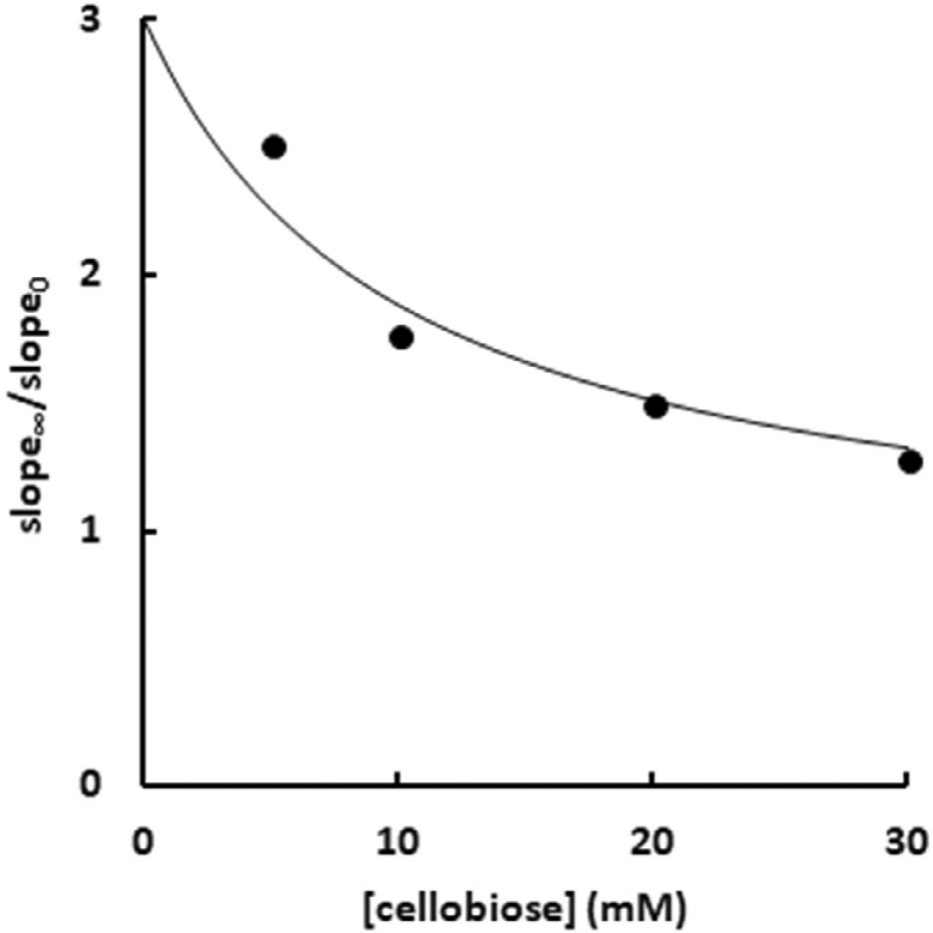
The ratio slope_∞_/slope_0_ for different cellobiose concentrations. The slope_∞_ and slope_0_ data were extracted from Fig. 7B (see Materials and methods for further details). The line represents the best fit of the data in Eqn (4) (*R* ^2^ = 0.96).

Considering that parameters α and *K*_j_ were previously determined (Table 2 and Fig. S11), Eqn (4) above was fitted into the slope_∞_/slope_0_ and [cellobiose] ([*J*]) data (extracted from Fig. 7B; see Materials and methods for further details) to estimate β as 3.9 ± 0.6 (Fig. 8).

This β estimate, higher than 1, but lower than infinite, indicates a mutual hindrance effect on the cellobiose and imidazole binding, but they are not mutually exclusive ligands. Moreover, β is similar to the α, previously determined from the imidazole inhibitory effect upon the substrate hydrolysis (Table 2). Thus, both approaches, the inhibition of the substrate hydrolysis with imidazole and the competition between imidazole and a second inhibitor (Figs 4, 5, 6, 7, 8), are reporting the same hindering effect between cellobiose and imidazole. Therefore, recalling that cellobiose definitely interacts with the active site, both experiments showed unequivocally that imidazole also binds in the Sfβgly active site.

**Fig. 9:**
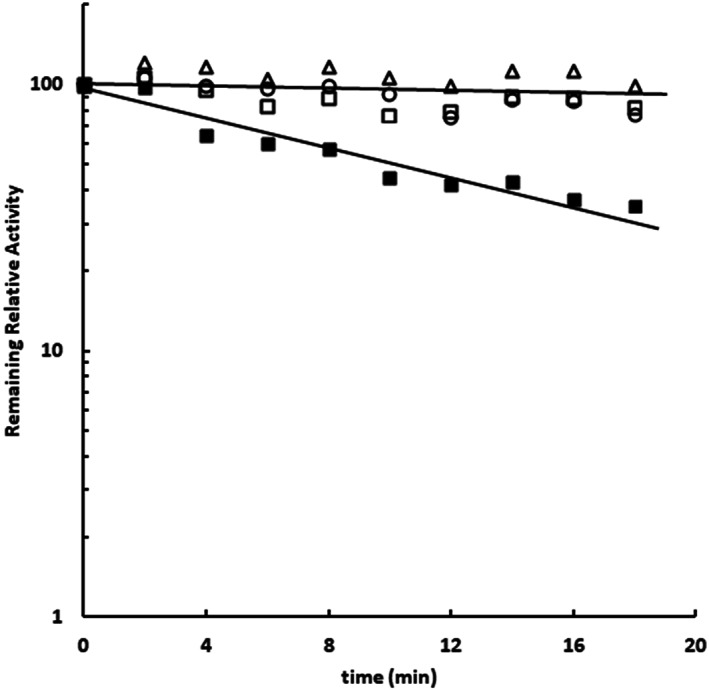
Inactivation of Sfβgly with carbodiimide and glycine ethyl ester (GEE). The inactivation was performed in the presence of 40 mm cellobiose (∆), 60 mm imidazole (○), and without any ligand (■). A control experiment, containing only the enzyme prepared in 100 mm phosphate buffer pH 6.0, was also performed (□). The 12 mm EDC and 40 mm GEE were prepared in 100 mm phosphate buffer pH 6.0. Reactions were performed at 30 °C. The remaining activity was determined by using 15 mm *p*‐nitrophenyl β‐glucoside as substrate. Substrate and ligands were also prepared in 100 mm phosphate buffer pH 6.0. Activity assays were performed at 30 °C. The *k*_obs_ of the inactivation reaction was calculated based on the line slope.

Finally, a third approach was devised to test the imidazole binding in the Sfβgly active site. Carboxylic groups from the side chain of glutamic acid residues can react with carbodiimides forming an ester that promptly reacts with primary amines at acidic pH [27]. The catalytic activity of the GH1 β‐glucosidases depends on two glutamic acid residues within their active site. For Sfβgly these residues are E187 and E399 [6]. Hence, 1‐ethyl‐3‐(dimethylamino‐propyl) carbodiimide (EDC) can prompt the reaction of these two E residues with glycine ethyl ester (GEE), converting their side chains in an amide and resulting in the Sfβgly inactivation [27]. The kinetics of the Sfβgly inactivation in the presence of 12 mm EDC and 40 mm GEE was determined (Fig. 9) showing an observable rate constant (*k*_obs_) of 0.024 min^−1^. A control reaction containing only the enzyme showed that Sfβgly is stable in those conditions. Interestingly, the addition of 40 mm cellobiose (4*K*_i_) to the reaction mix abolished the enzyme inactivation promoted by EDC, suggesting that the cellobiose binding hinders the EDC and GEE accesses to the active site, protecting the enzyme from inactivation. Similarly, the addition of 60 mm imidazole (4*K*_i_) to that modification reaction also protected Sfβgly from inactivation (Fig. 9). Hence, the saturating concentration of both, cellobiose and imidazole, hampers the access of EDC and GEE to the Sfβgly catalytic residues, confirming that imidazole binding spot is within the Sfβgly active site.

**Fig. 10:**
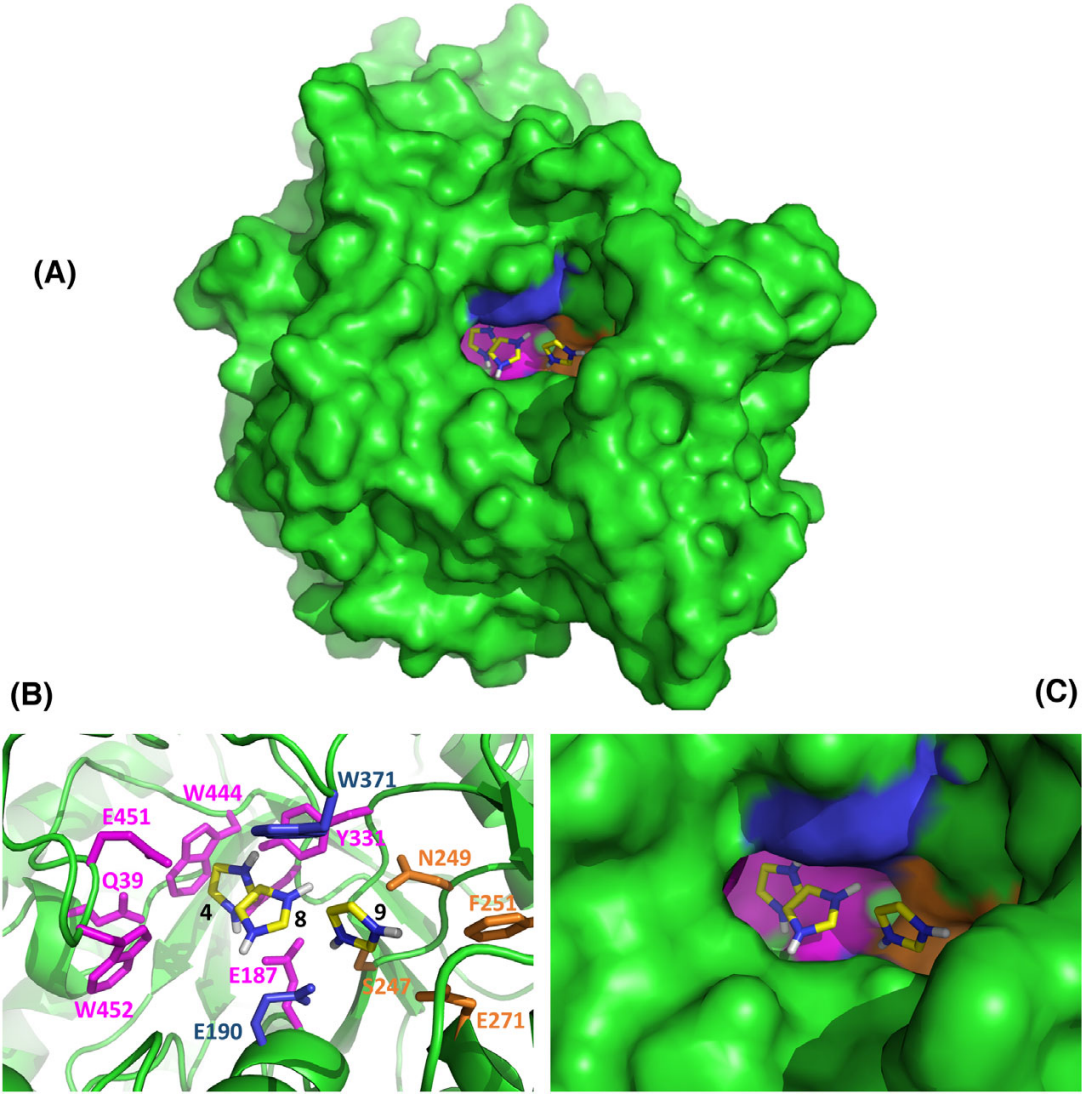
Imidazole binding within different regions of the Sfβgly active site. (A) General perspective of the Sfβgly molecular surface showing the active site niche containing the imidazole docking solutions 4, 8, and 9 (Table 1). These solutions illustrate the binding in the subsite −1 (pink), subsite +1 (blue), and the pocket in the active site entrance (orange). (B) Detailed structural features of the imidazole docking solutions 4, 8, and 9 within the subsite −1 (pink), subsite +1 (blue), and the pocket in the active site entrance (orange), respectively. Residues shown as sticks are within 3.5 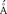 from the N_1_ or N_3_ of the imidazole. (C) Molecular surface of the active site niche showing the imidazole docking solutions 4, 8, and 9 within the subsite −1 (pink), subsite +1 (blue), and the pocket in the active site entrance (orange), respectively. Panels B and C are different representations of the same view.

Therefore, the different approaches presented above converged showing that imidazole binds to the Sfβgly active site and reduces the substrate affinity. Taking into consideration the indications of the molecular docking initially presented (Fig. 1; Table 1), the imidazole could be occupying the inner portions of the active site or even its entrance (Figs 1B–D and 10A). These three binding spots are properly represented in the docking solutions 4, 8, and 9 (Fig. 10B). Nevertheless, the imidazole docking in the −1 subsite seems too restrictive for the simultaneous imidazole and substrate interactions (Figs 1B and 10B,C). Conversely, the imidazole binding in the entrance of the active site, a wider region involving the +1 subsite and the lateral pocket (Figs 1C,D and 10B,C), could weaken the substrate binding but keep the enzyme equally active once the substrate–enzyme complex were formed, that is, it would result in a productive ESI complex (Fig. 4). Indeed, the structural superimposition of the active sites of the Sfβgly with the imidazole docking solutions 4, 8, and 9 and GH1 β‐glucosidases complexed with a substrate (PDB 3AI0, 2O9R, and 2Z1S; Fig. S17), suggests that the imidazole binding within the lateral pocket in the entrance of the active site (Fig. 10B,C) is compatible with an ESI complex. Hence, this binding spot, the pocket nearby the active site opening (docking solutions 9 and 10 in Table 1; Figs 1D and 10B,C), is an attractive hypothesis to explain our results.

Finally, considering that residues forming the active site are conserved among the GH1 β‐glucosidases, this imidazole inhibition may be widespread within this enzyme family. Hence, this information should be taken into account particularly in the characterization of the catalytic activity and enzyme kinetic parameters of recombinant GH1 β‐glucosidases purified using imidazole.

## Materials and methods

### Production and purification of the recombinant Sfβgly

The recombinant Sfβgly was produced in BL21(DE3) bacteria using the expression vector pET46 as previously described in [23]. Following that, recombinant Sfβgly was purified by affinity to the Ni‐NTA agarose resin (Qiagen) as also previously described in [23]. The homogeneity of the recombinant Sfβgly was checked in SDS/PAGE [28] (Fig. S1). Protein concentration was determined by the bicinchoninic acid method [29]. The purified Sfβgly sample was submitted to buffer exchange by using PD Minitrap G‐25 columns (Cytiva, Marlborough, MA, USA). After that, this final sample in 100 mm phosphate buffer pH 6 was stored at 4 °C.

### Determination of the Sfβgly catalytic activity

The Sfβgly hydrolysis activity upon *p*‐nitrophenyl β‐glucoside was determined in reactions (100 μL) performed at 30 °C and interrupted by the addition of 0.5 m Na_2_CO_3_ (100 μL). The product (*p*‐nitrophenolate) formation was followed by absorbance at 415 nm. Absorbance was converted into mol by using standard curves prepared in the same conditions as the enzymatic reactions. Briefly, different amounts (nmols) of *p*‐nitrophenol prepared in 100 mm phosphate buffer pH 6 (final volume of 100 μL) were combined with 0.5 m Na_2_CO_3_ (100 μL). Their absorbance at 415 nm and amounts (nmols) of *p*‐nitrophenol were plotted and submitted to linear regression. The line equation was used to convert the absorbance into mol of *p*‐nitrophenol (product) formed along the enzyme assays.

The Sfβgly hydrolysis activity upon cellobiose and cellotetraose was determined by following the formation of glucose. Reactions (100 μL) were performed at 30 °C and interrupted by boiling (3 min). Glucose formation was detected with the Trinder kit (Analisa, Belo Horizonte, Brazil). This kit is based on the oxidation of glucose catalyzed by the enzyme glucose oxidase, which also produces hydrogen peroxide. That last product reacts with 4‐aminoantipyrine and phenol resulting in a dye that absorbs at 490 nm. Standard curves for glucose quantification were prepared by combining different amounts (nmols) of glucose (final volume of 100 μL) prepared in 100 mm phosphate buffer pH 6 with the reaction solution (150 μL) of Trinder kit. After 10 min at 30 °C their absorbance at 490 nm was determined. The amounts (nmols) of glucose and absorbance data were plotted and submitted to linear regression. The line equation was used to convert the absorbance at 490 nm into mol of glucose (product) formed along the enzyme assays.

The occurrence of transglycosylation reactions was evaluated by following the ratio of the two products, *p*‐nitrophenolate and glucose, generated from 15 mm *p*‐nitrophenyl β‐glucoside. Reactions (100 μL) were performed at 30 °C and interrupted by boiling (3 min). The reaction mix was divided into two equal aliquots, which were used for the product detection. The *p*‐nitrophenolate and glucose were determined as indicated above.

Initial rates were determined based on the line slope in the [product] *vs* time plots. Linear regression was used to evaluate the data. Correlation coefficients higher than 0.95 were accepted.

The Sfβgly hydrolysis activity upon 15 mm *p*‐nitrophenyl β‐glucoside was determined in the absence and presence of 150 mm imidazole. Assays were performed as described above. Substrate and imidazole were prepared in 100 mm phosphate buffer pH 6.

The effect of the imidazole on the Sfβly stability was evaluated by incubating the enzyme in the presence and absence of 150 mm imidazole for 18 h at 30 °C. After that, the imidazole was washed away with 50 volumes of 100 mm phosphate buffer pH 6 by using centrifugal filter devices (Amicon Ultracel‐10K, Millipore, Burlington, MA, USA). Next, the enzyme activity of these two samples (after removal of imidazole) was determined by using 15 mm *p*‐nitrophenyl β‐glucoside as previously described. Substrate and imidazole were prepared in 100 mm phosphate buffer pH 6.0.

In order to prevent changes in the buffer pH, buffering reagents were previously combined with imidazole and/or NaCl in the appropriate proportion to attain the concentration and pH of interest. Next, these reagents were dissolved, and after that, the buffer pH was set to 6.0. NaCl was added to the buffer in reactions without imidazole to normalize the ionic strength.

### Inhibition of the Sfβgly activity with imidazole

The initial rate of hydrolysis of at least 10 different substrate concentrations was determined in the presence of at least five different imidazole concentrations (0–120 mm). Substrates were *p*‐nitrophenyl β‐glucoside (0.4–10 mm), cellobiose (0.6–16 mm), and cellotetraose (0.2–2 mm). Reactions were performed at 30 °C. Substrates and inhibitors were prepared in 100 mm phosphate buffer pH 6.0. The product formation (*p*‐nitrophenolate and glucose) was detected as described above. NaCl was added to the inhibitor solution to normalize the ionic strengths among reactions, taking 120 mm imidazole as a referential. Initial rates were determined based on three product quantifications. Each complete experiment was performed with three different enzyme samples. Initial rates and substrate concentration data were analyzed using Lineweaver–Burk plots. Line slopes and intercepts correlation with inhibitor concentration was used to identify the inhibition mechanism [26]. Data were submitted to linear regression analysis. Fittings were accepted when showing linear correlation coefficients higher than 0.9. Kinetic parameters (*K*_i_ and α) were expressed as median and standard deviation resulting from three independent experiments.

### Inhibition of the Sfβgly activity with cellobiose

The initial rate of hydrolysis of at least 10 different *p*‐nitrophenyl β‐glucoside (0.4–10 mm) concentrations was determined in the presence of five different cellobiose concentrations (0–45 mm). Reactions were performed at 30 °C. *p*‐nitrophenyl β‐glucoside and cellobiose were prepared in 100 mm phosphate buffer pH 6.0. The product formation (*p*‐nitrophenolate) was detected as described above. Initial rates were determined based on three product quantifications using the same enzyme sample. Initial rates and substrate concentration data were analyzed using Lineweaver–Burk plots. Data were submitted to linear regression analysis. Fittings were accepted when showing linear correlation coefficients higher than 0.9. Line slopes and intercepts correlations with cellobiose concentration were used to identify the inhibition mechanism [26].

### Simultaneous inhibition of Sfβgly activity with imidazole and a competitive inhibitor

The initial rate of hydrolysis of at least 10 different *p*‐nitrophenyl β‐glucoside concentrations (0.4–10 mm) was determined in the presence of five different imidazole concentrations (0–120 mm). Each of these experiments was performed in the presence of fixed cellobiose concentrations (5–30 mm), which was used as a competitive inhibitor. Reactions were performed at 30 °C. The product formation (*p*‐nitrophenolate) was detected as described above. NaCl was added to the inhibitor solution to normalize the ionic strengths among reactions, taking 120 mm imidazole as a referential. Initial rates of the *p*‐nitrophenyl β‐glucoside hydrolysis were determined based on three product quantifications using the same enzyme sample. Initial rate condition also applies to the cellobiose hydrolysis. So, both *p*‐nitrophenyl β‐glucoside and cellobiose concentrations are considered constant in this experiment. Initial rates and substrate concentration data were analyzed using Lineweaver–Burk plots. The correlations between line slopes and imidazole concentration were used to evaluate the cellobiose effect on the inhibition mechanism. The slope in the absence of imidazole (slope_0_) was estimated from the intercept of those plots. On the contrary, the slope at infinite imidazole concentration (slope_∞_) was estimated from the intercept of the 1/line slope *vs* [imidazole]. Equation (4) was fitted into the slope_∞_/slope_0_ and [cellobiose] by using nonlinear regression analysis in the Origin 2019 software.

### Inactivation of Sfβgly with carbodiimide

Inactivation reactions were performed by incubating Sfβgly in the presence of 12 mm EDC and 40 mm GEE at 30 °C [27]. Samples of the reaction mix were removed after different incubation times, combined with 100 mm citrate–phosphate buffer pH 6 to react with and remove the EDC excess. Next, these samples were employed to determine the remaining enzyme activity upon 15 mm *p*‐nitrophenyl β‐glucoside as described above. A sample of Sfβgly was incubated in the same conditions, but in the absence of EDC and GEE, to evaluate the enzyme stability. 40 mm cellobiose and 60 mm imidazole were separately added to the inactivation reaction to evaluate their enzyme protection effect. The enzyme inactivation was analyzed as a pseudo‐first‐order reaction by plotting the log(relative remaining activity) *vs* time. Hence, the observable rate constant of the enzyme inactivation (*k*_obs_) was estimated from the slope of that line. Substrate, EDC and GEE, cellobiose, and imidazole were prepared in 100 mm phosphate buffer pH 6.0. Data were submitted to linear regression analysis. Fittings were accepted when showing linear correlation coefficients higher than 0.9.

### Computational docking between imidazole and Sfβgly

Sfβgly initial coordinates were taken from the PDB entry 5CG0. The imidazole 3D coordinates were generated in Gabedit v. 2.5.1 [30]. The addition of hydrogen atoms, and deletion of water molecules, ions, and other molecules from the protein initial structure were carried out using Chimera v. 1.14 [31], as well as the grid box coordinates covering all protein atoms, as previously described [32]. Docking of imidazole and Sfβgly was performed using AutoDock Vina v. 1.1.2 [33] launched from Chimera searching the 10 best models. The imidazole was docked in the mono‐protonated (+1 charge) state. The Sfβgly–imidazole complexes representing the different docking solutions were visualized by using the pymol v 0.99 software (Schrödinger LLC, New York, NY, USA).

The crystallographic structures of the β‐glucosidases from *Neotermes koshunensis* and *Paenibacillus polymyxa* (PDB ID 3AI0, 2O9R, 2Z1S, respectively) containing the substrates NPbglc, thiocellobiose and C4 were structurally superposed to the Sfβgly (PDB ID 5CG0). Next, the structure of the β‐glucosidases from *N. koshunensis* and *P. polymyxa* were removed leaving only the substrate overlaid on the Sfβgly active site niche. The superimpositions were prepared and visualized using the pymol v 0.99 software.

## Conflict of interest

The authors declare no conflict of interest.

## Author contributions

RSC, RKS, and SRM planned the experiments. RSC and MARP performed the experiments. RSC, FAMO, MARP, RKS, and SRM analyzed the data. RKS and SRM contributed to the resources. RSC, FAMO, MARP, RKS, and SRM involved in writing—original draft, and review and editing.

## Supporting information


**Fig. S1.** SDS/PAGE of purified recombinant Sfβgly.

**Fig. S2.** Determination of the mechanism of Sfβgly inhibition by imidazole. The substrate is *p*‐nitrophenyl β‐glucoside. Enzyme sample number: 1.

**Fig. S3.** Determination of the mechanism of Sfβgly inhibition by imidazole. The substrate is *p*‐nitrophenyl β‐glucoside. Enzyme sample number: 2.

**Fig. S4.** Determination of the mechanism of Sfβgly inhibition by imidazole. The substrate is *p*‐nitrophenyl β‐glucoside. Enzyme sample number: 3.

**Fig. S5.** Determination of the mechanism of Sfβgly inhibition by imidazole. The substrate is cellobiose. Enzyme sample number: 1.

**Fig. S6.** Determination of the mechanism of Sfβgly inhibition by imidazole. The substrate is cellobiose. Enzyme sample number: 2.

**Fig. S7.** Determination of the mechanism of Sfβgly inhibition by imidazole. The substrate is cellobiose. Enzyme sample number: 3.

**Fig. S8.** Determination of the mechanism of Sfβgly inhibition by imidazole. The substrate is cellotetraose. Enzyme sample number: 1.

**Fig. S9.** Determination of the mechanism of Sfβgly inhibition by imidazole. The substrate is cellotetraose. Enzyme sample number: 3.

**Fig. S10.** Determination of the mechanism of Sfβgly inhibition by imidazole. The substrate is cellotetraose. Enzyme sample number: 2.

**Fig. S11.** Determination of the mechanism of Sfβgly inhibition by cellobiose. The substrate is *p*‐nitrophenyl β‐glucoside.

**Fig. S12.** Determination of the mechanism of Sfβgly inhibition by imidazole in the presence of 5 mM cellobiose. The substrate is *p*‐nitrophenyl β‐glucoside.

**Fig. S13.** Determination of the mechanism of Sfβgly inhibition by imidazole in the presence of 10 mM cellobiose. The substrate is *p*‐nitrophenyl β‐glucoside.

**Fig. S14.** Determination of the mechanism of Sfβgly inhibition by imidazole in the presence of 20 mM cellobiose. The substrate is *p*‐nitrophenyl β‐glucoside.

**Fig. S15.** Determination of the mechanism of Sfβgly inhibition by imidazole in the presence of 30 mM cellobiose. The substrate is *p*‐nitrophenyl β‐glucoside.

**Fig. S16.** Deduction of the rate equation describing the simultaneous effect of a partial competitive and a competitive inhibitor on the enzymatic hydrolysis of the substrate.

**Fig. S17.** Imidazole and substrate binding within the Sfβgly active site.

**Table S1.** Kinetic parameters for the hydrolysis of different substrates by Sfβgly.

## Data Availability

The data that support the findings of this study are available in the figures, tables, and the supplementary material of this article.
