# Supplementary figures and images for: Mechanism of imidazole inhibition of a GH1 β‐glucosidase
Source: FEBS Open Bio. 2023 Mar 25;13(5):912–25. doi: 10.1002/2211-5463.13595 (PMC10153361; doi:10.1002/2211-5463.13595)

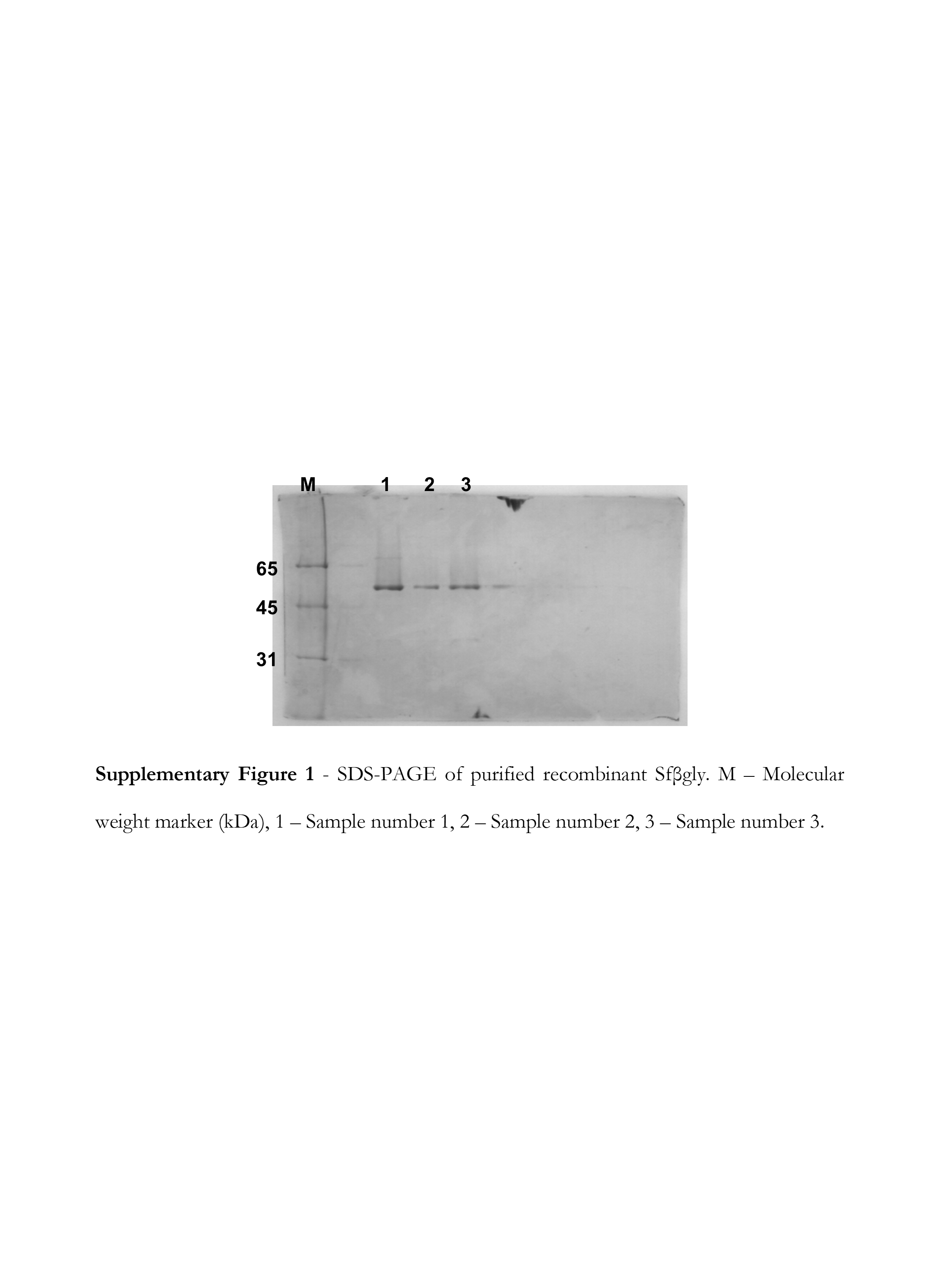

Supplement: Supplementary file 1 — Fig. S1. SDS/PAGE of purified recombinant Sfβgly. [file FEB4-13-912-s012.tif]

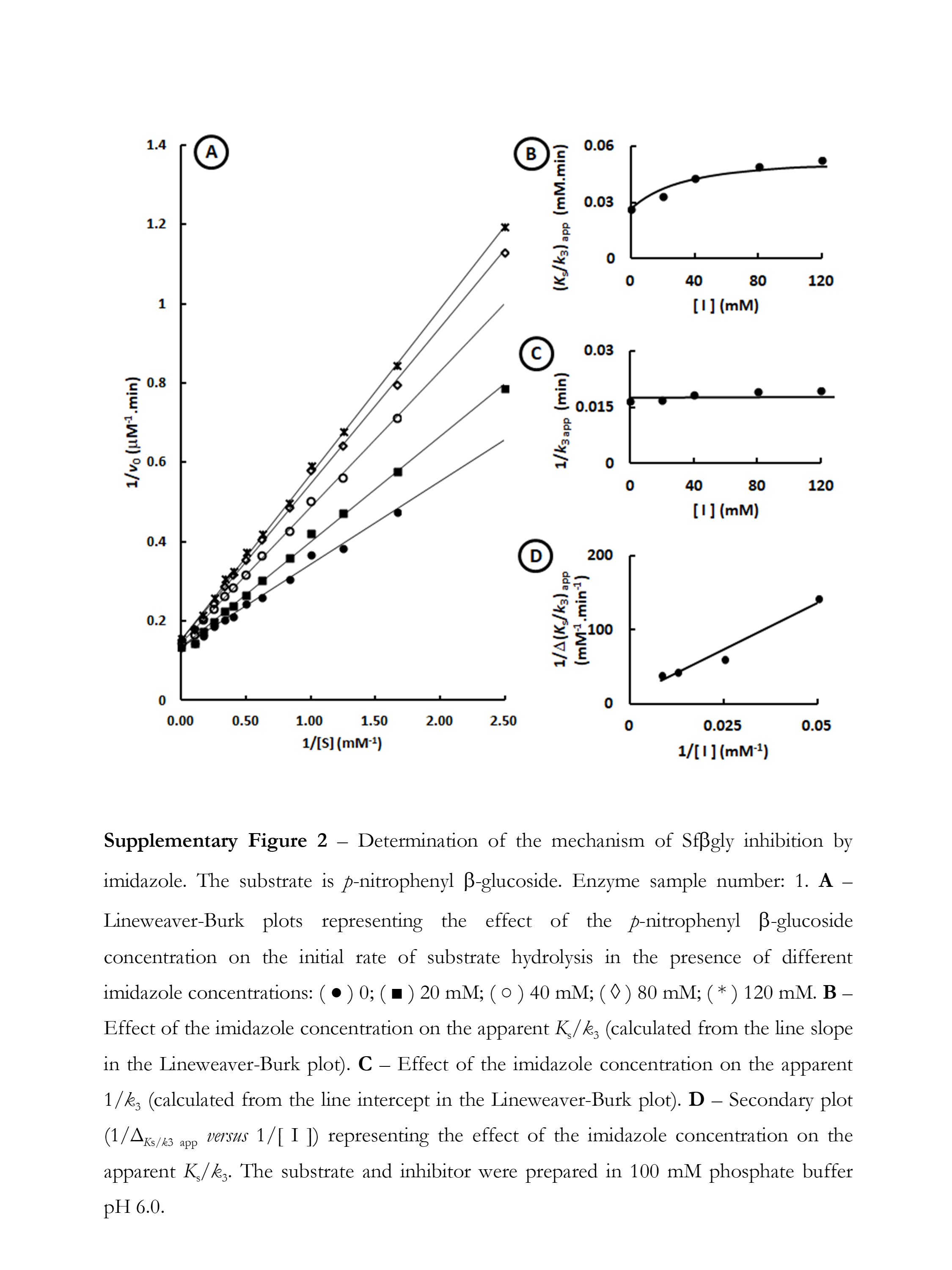

Supplement: Supplementary file 2 — Fig. S2. Determination of the mechanism of Sfβgly inhibition by imidazole. The substrate is p‐nitrophenyl β‐glucoside. Enzyme sample number: 1. [file FEB4-13-912-s003.tif]

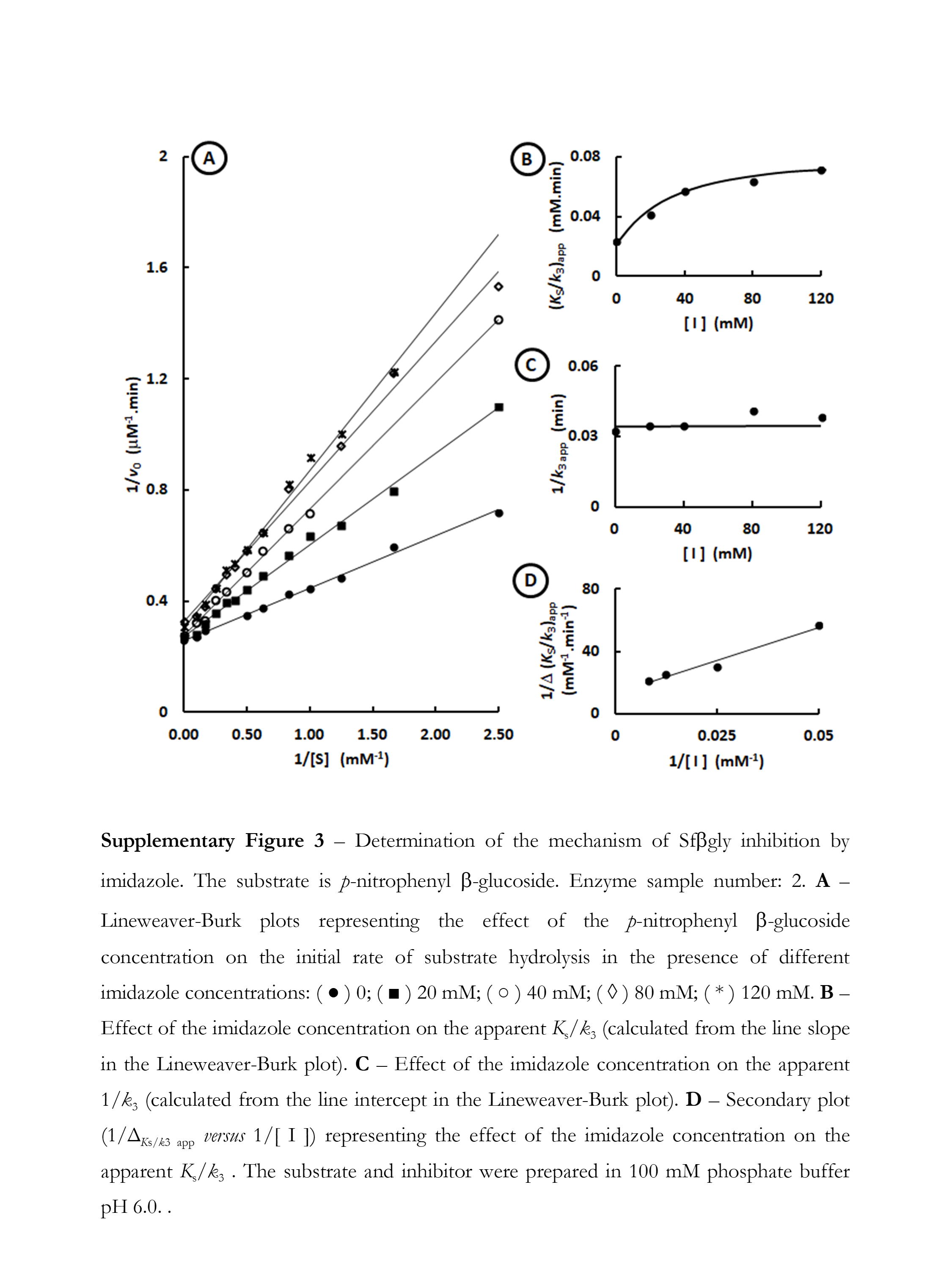

Supplement: Supplementary file 3 — Fig. S3. Determination of the mechanism of Sfβgly inhibition by imidazole. The substrate is p‐nitrophenyl β‐glucoside. Enzyme sample number: 2. [file FEB4-13-912-s015.tif]

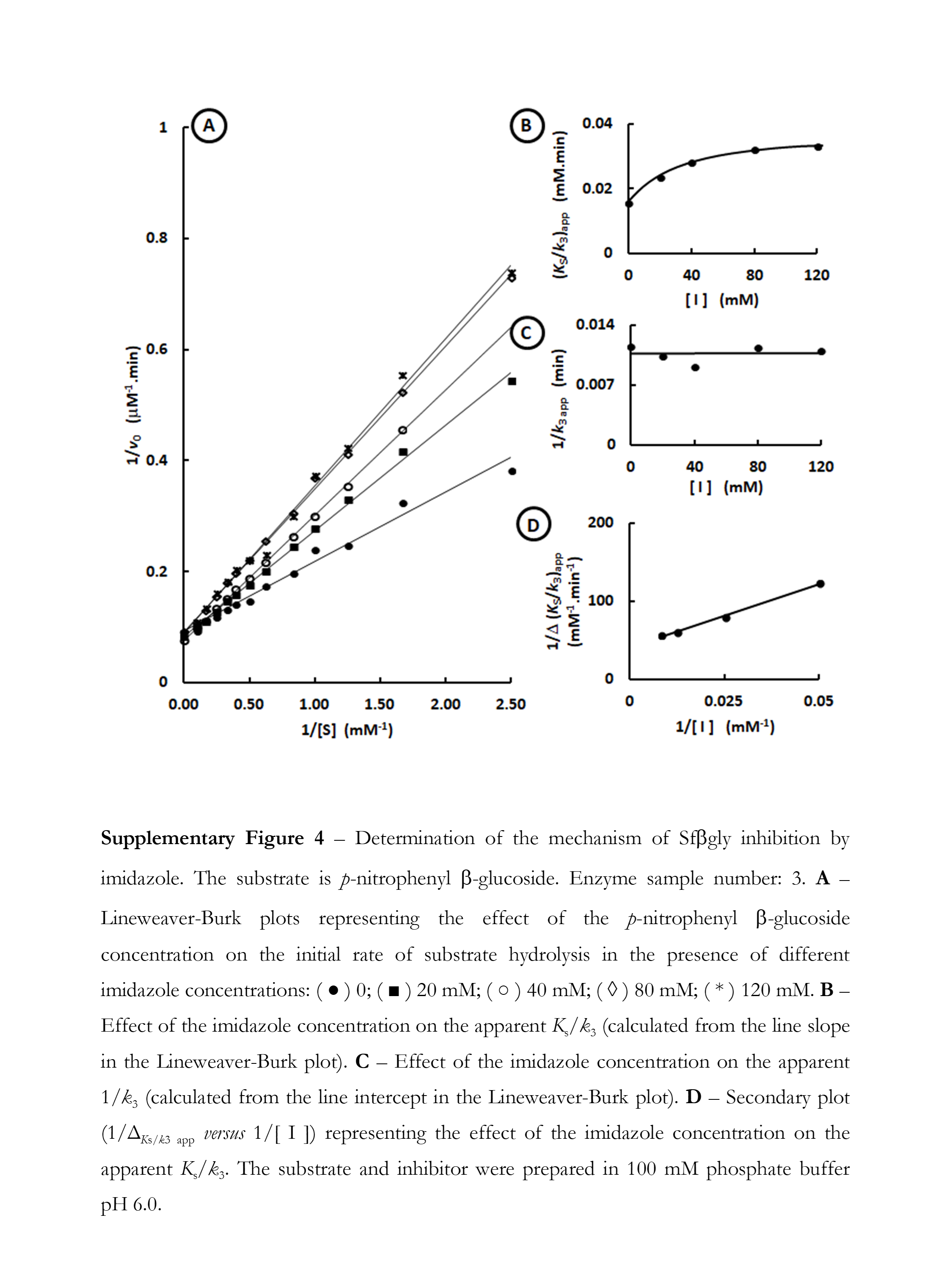

Supplement: Supplementary file 4 — Fig. S4. Determination of the mechanism of Sfβgly inhibition by imidazole. The substrate is p‐nitrophenyl β‐glucoside. Enzyme sample number: 3. [file FEB4-13-912-s002.tif]

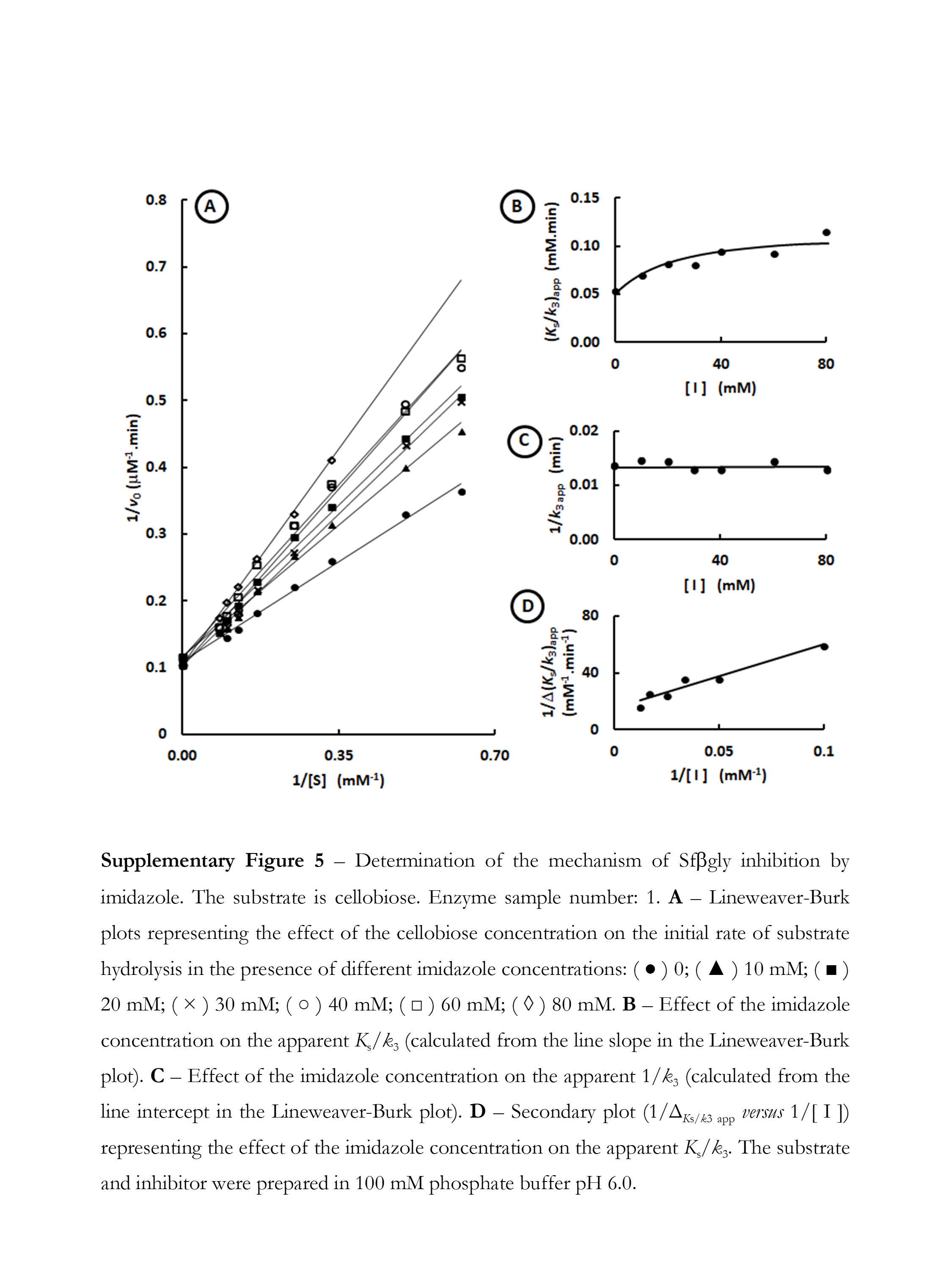

Supplement: Supplementary file 5 — Fig. S5. Determination of the mechanism of Sfβgly inhibition by imidazole. The substrate is cellobiose. Enzyme sample number: 1. [file FEB4-13-912-s001.tif]

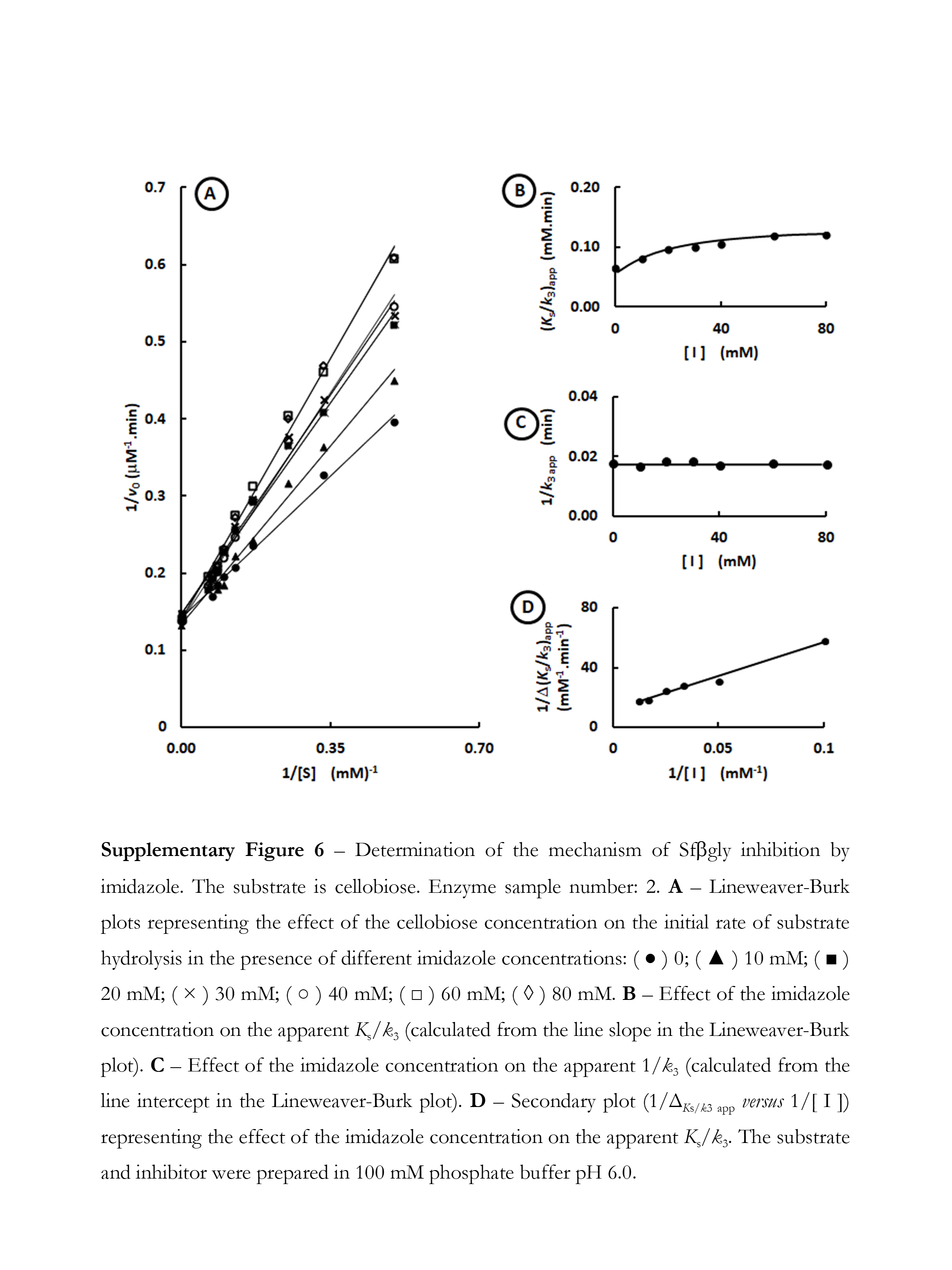

Supplement: Supplementary file 6 — Fig. S6. Determination of the mechanism of Sfβgly inhibition by imidazole. The substrate is cellobiose. Enzyme sample number: 2. [file FEB4-13-912-s018.tif]

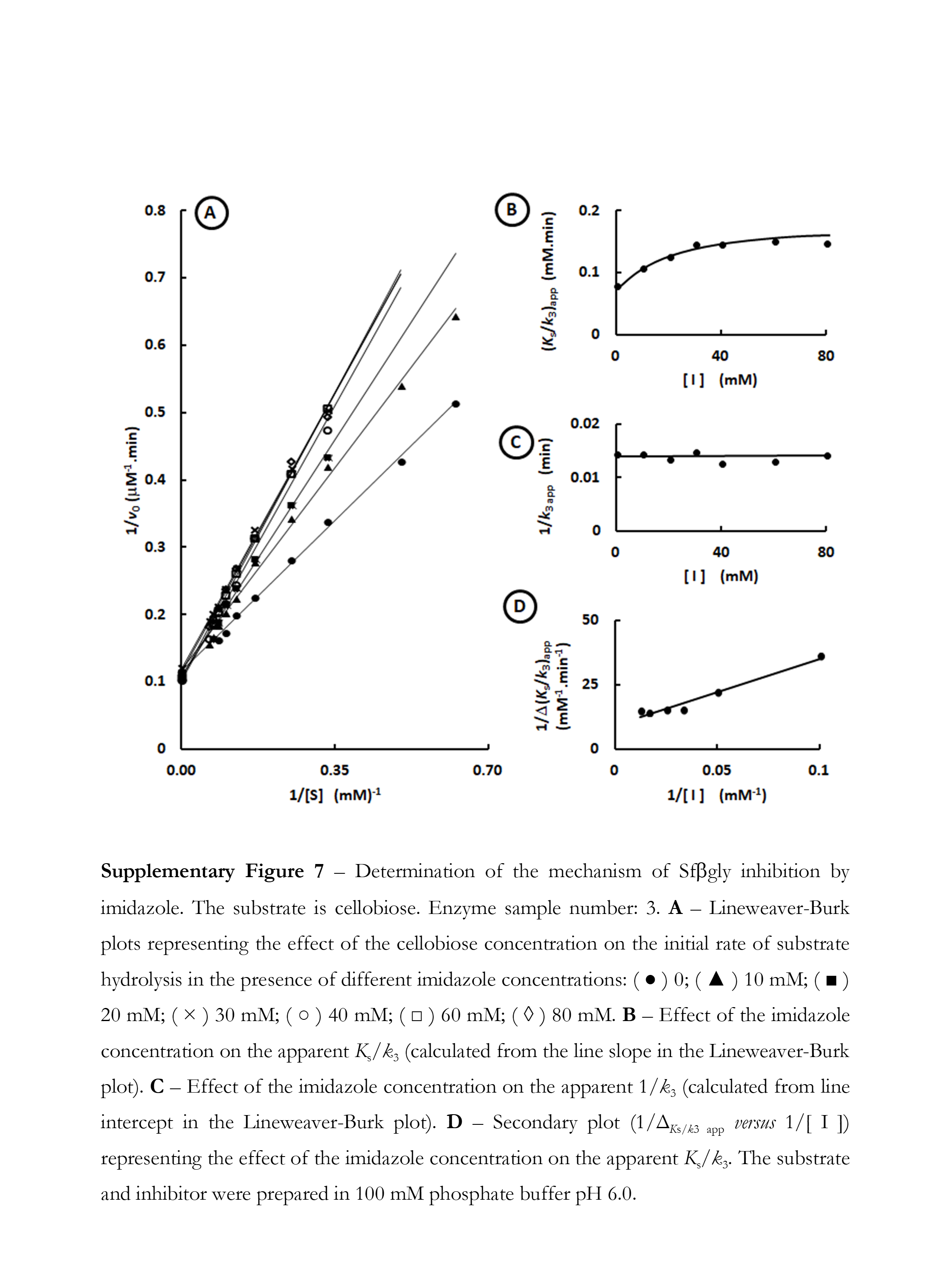

Supplement: Supplementary file 7 — Fig. S7. Determination of the mechanism of Sfβgly inhibition by imidazole. The substrate is cellobiose. Enzyme sample number: 3. [file FEB4-13-912-s006.tif]

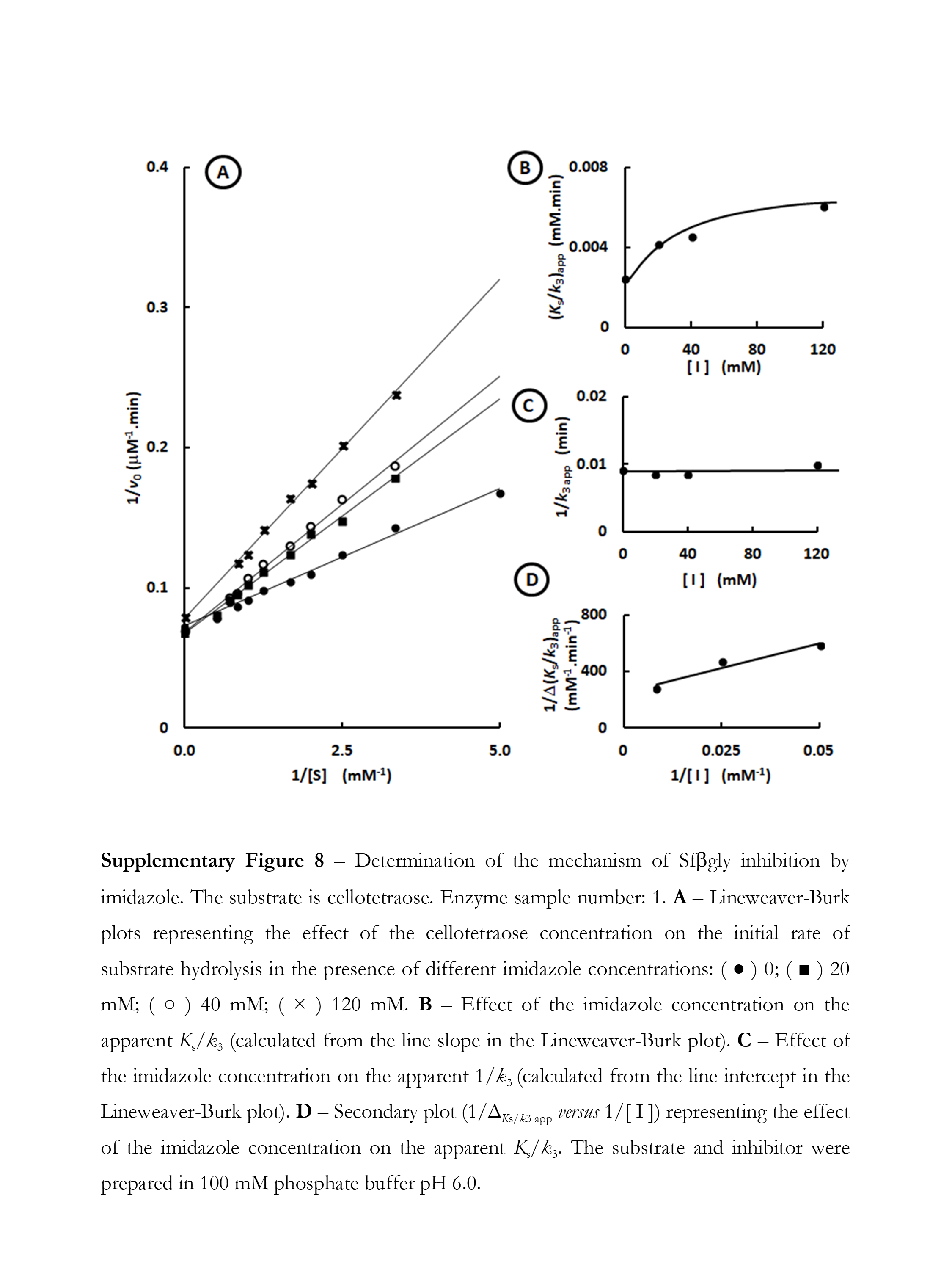

Supplement: Supplementary file 8 — Fig. S8. Determination of the mechanism of Sfβgly inhibition by imidazole. The substrate is cellotetraose. Enzyme sample number: 1. [file FEB4-13-912-s007.tif]

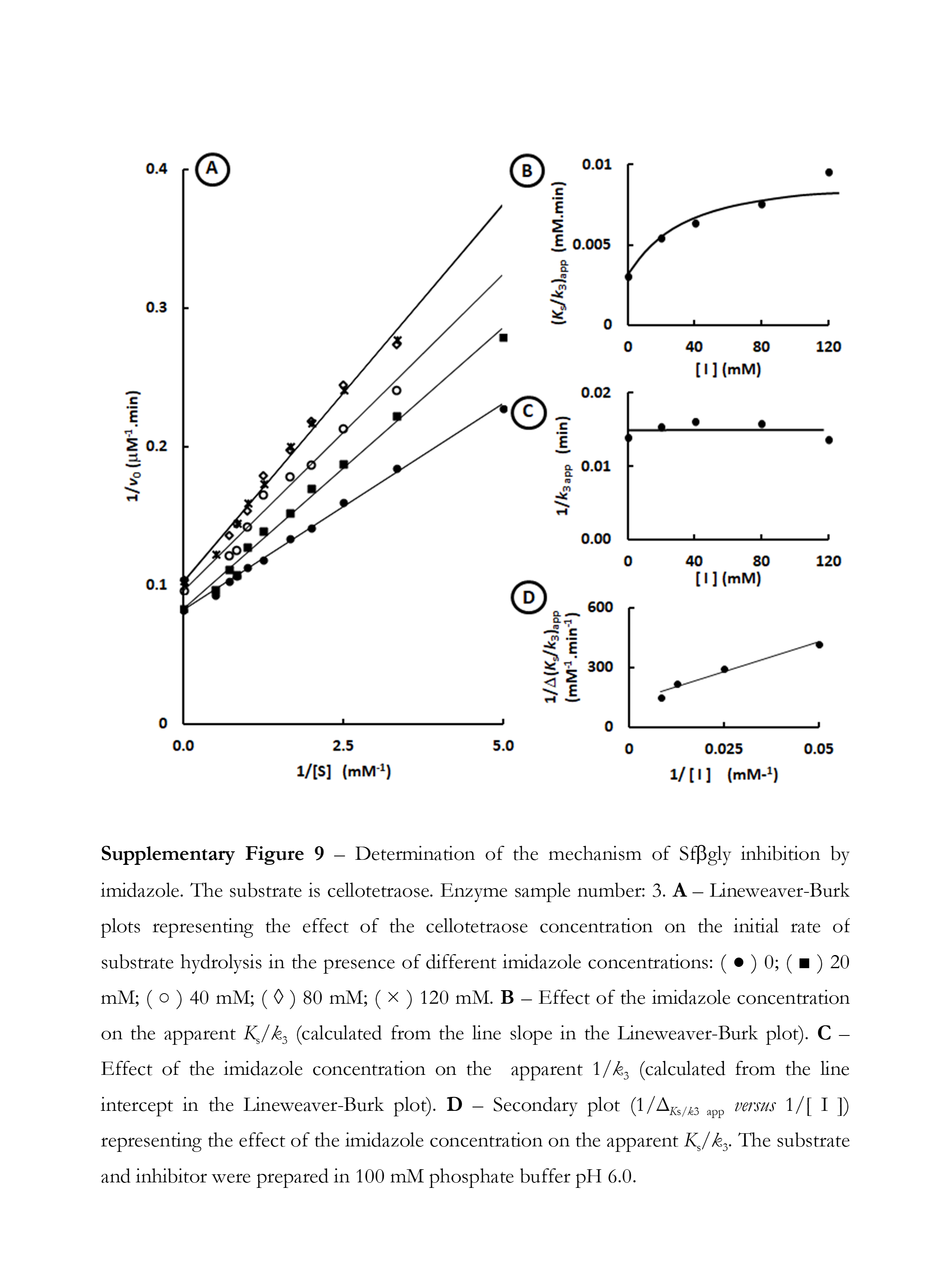

Supplement: Supplementary file 9 — Fig. S9. Determination of the mechanism of Sfβgly inhibition by imidazole. The substrate is cellotetraose. Enzyme sample number: 3. [file FEB4-13-912-s008.tif]

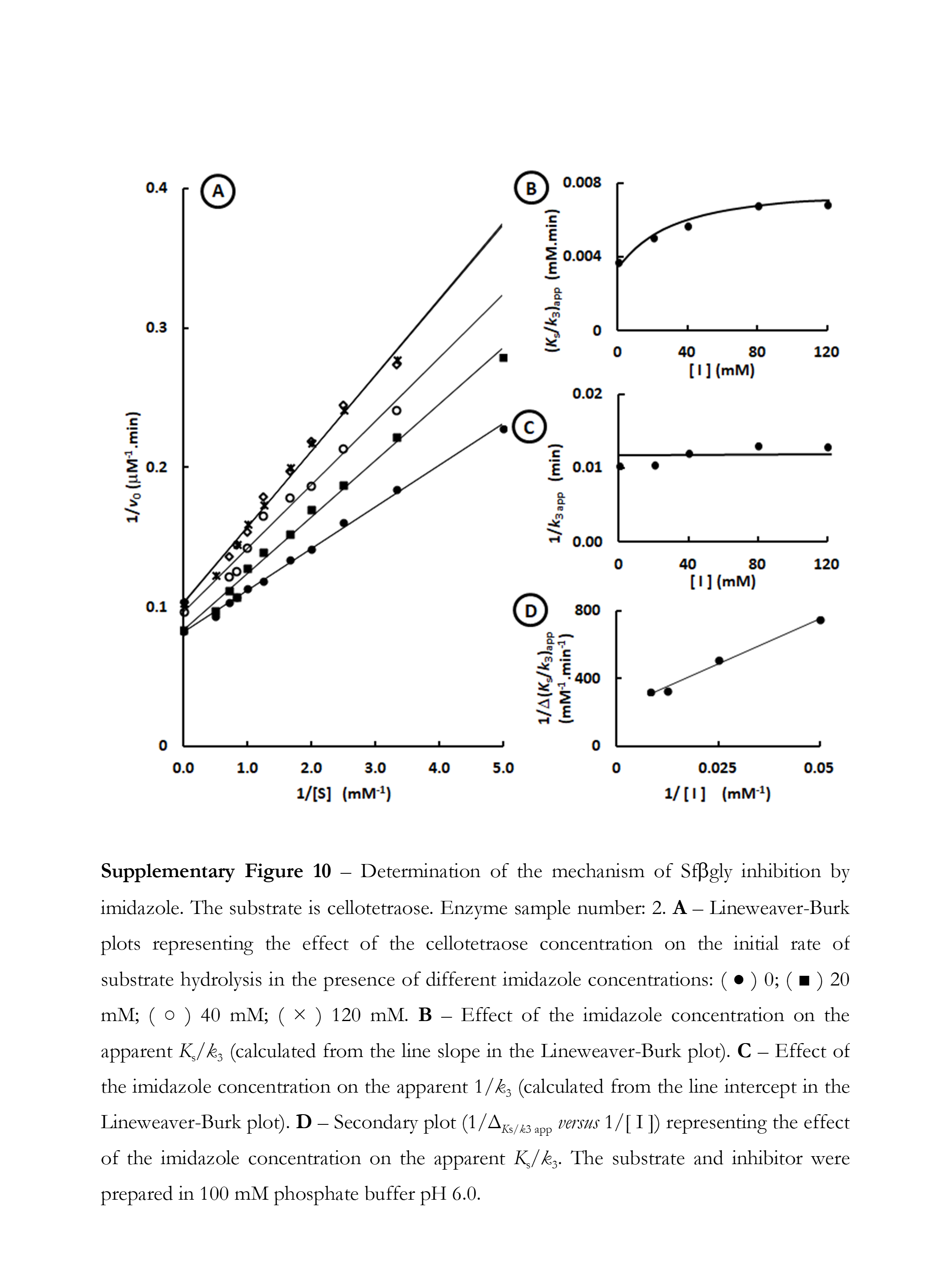

Supplement: Supplementary file 10 — Fig. S10. Determination of the mechanism of Sfβgly inhibition by imidazole. The substrate is cellotetraose. Enzyme sample number: 2. [file FEB4-13-912-s004.tif]

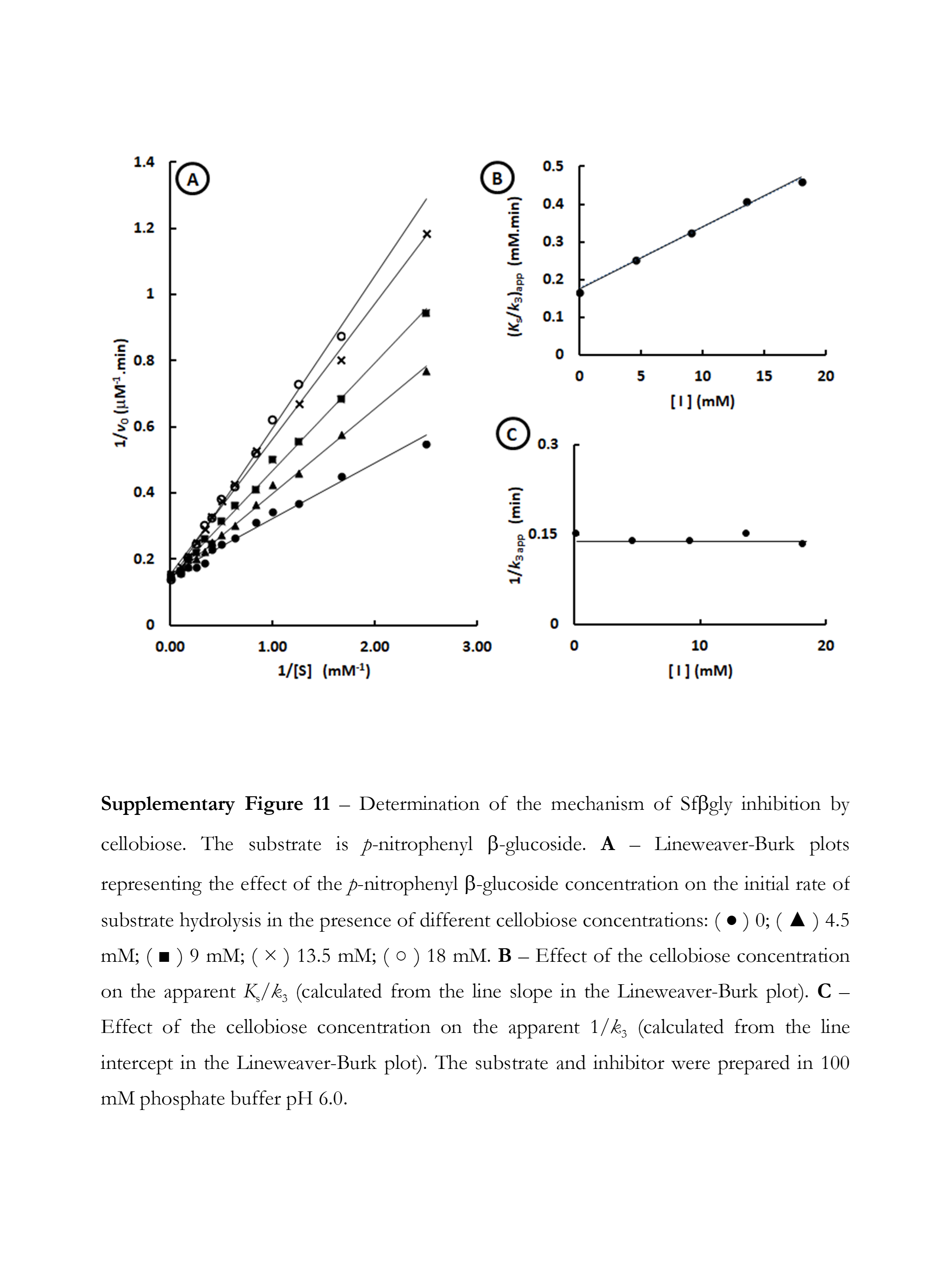

Supplement: Supplementary file 11 — Fig. S11. Determination of the mechanism of Sfβgly inhibition by cellobiose. The substrate is p‐nitrophenyl β‐glucoside. [file FEB4-13-912-s011.tif]

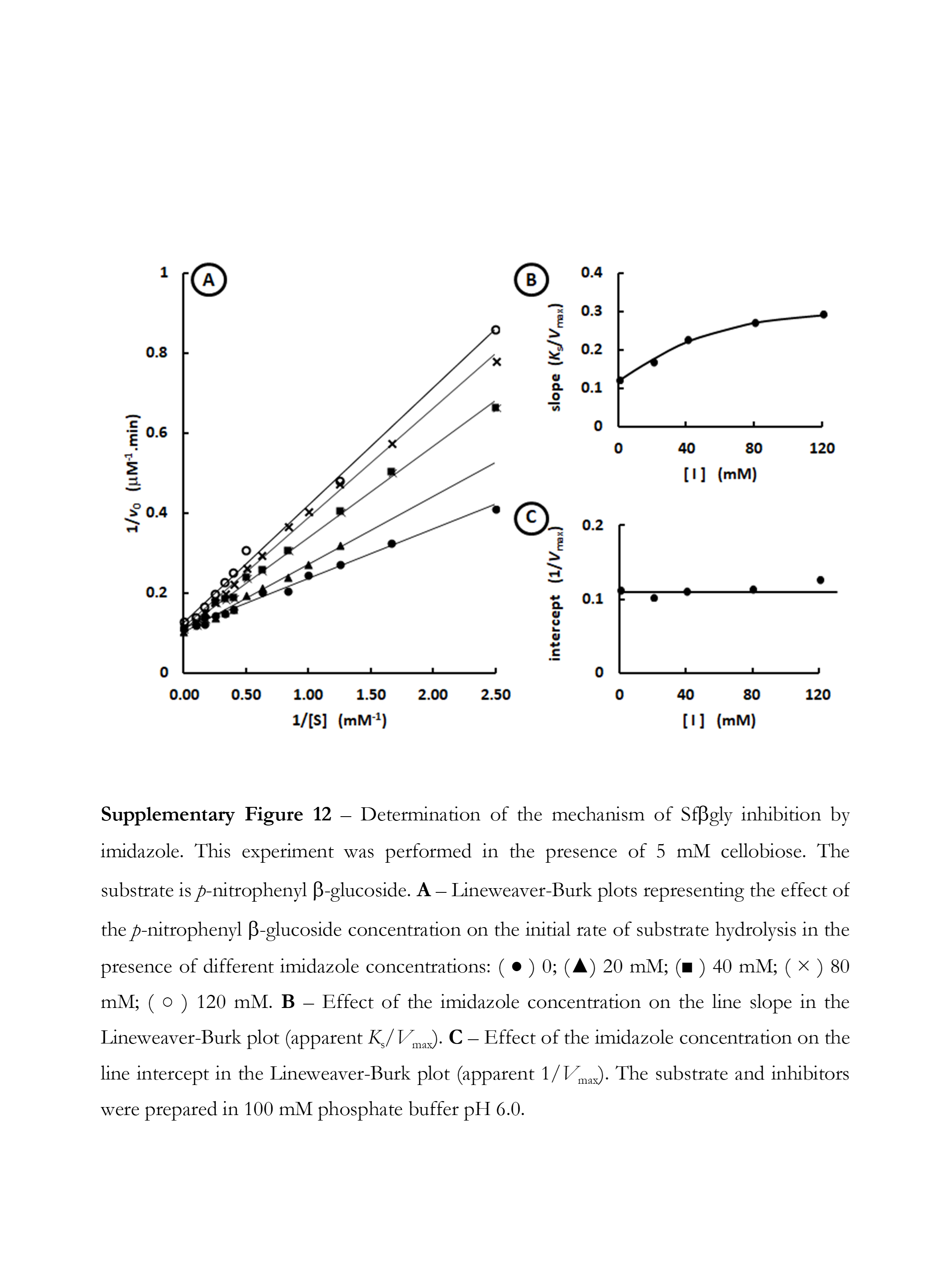

Supplement: Supplementary file 12 — Fig. S12. Determination of the mechanism of Sfβgly inhibition by imidazole in the presence of 5 mM cellobiose. The substrate is p‐nitrophenyl β‐glucoside. [file FEB4-13-912-s009.tif]

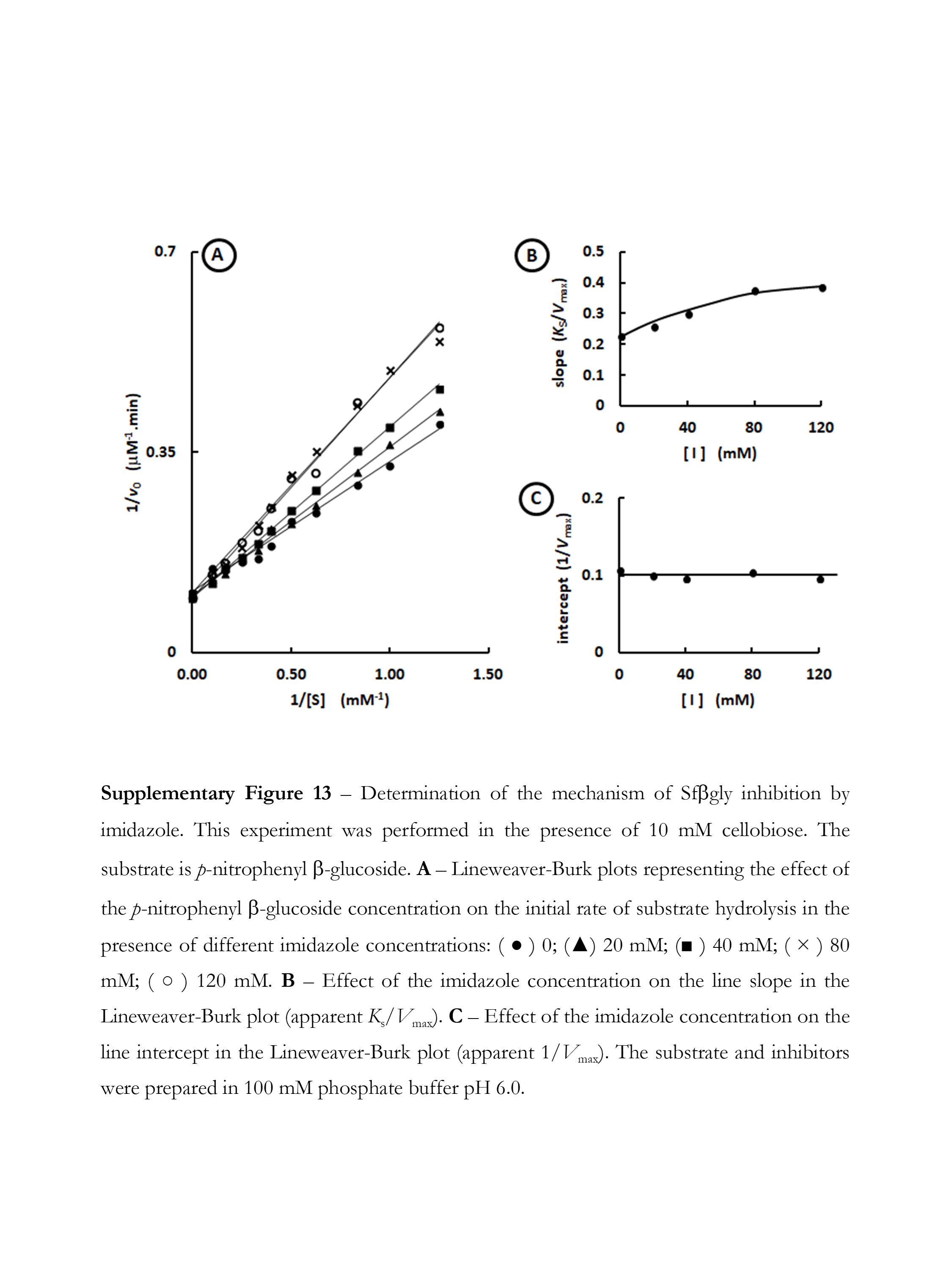

Supplement: Supplementary file 13 — Fig. S13. Determination of the mechanism of Sfβgly inhibition by imidazole in the presence of 10 mM cellobiose. The substrate is p‐nitrophenyl β‐glucoside. [file FEB4-13-912-s005.tif]

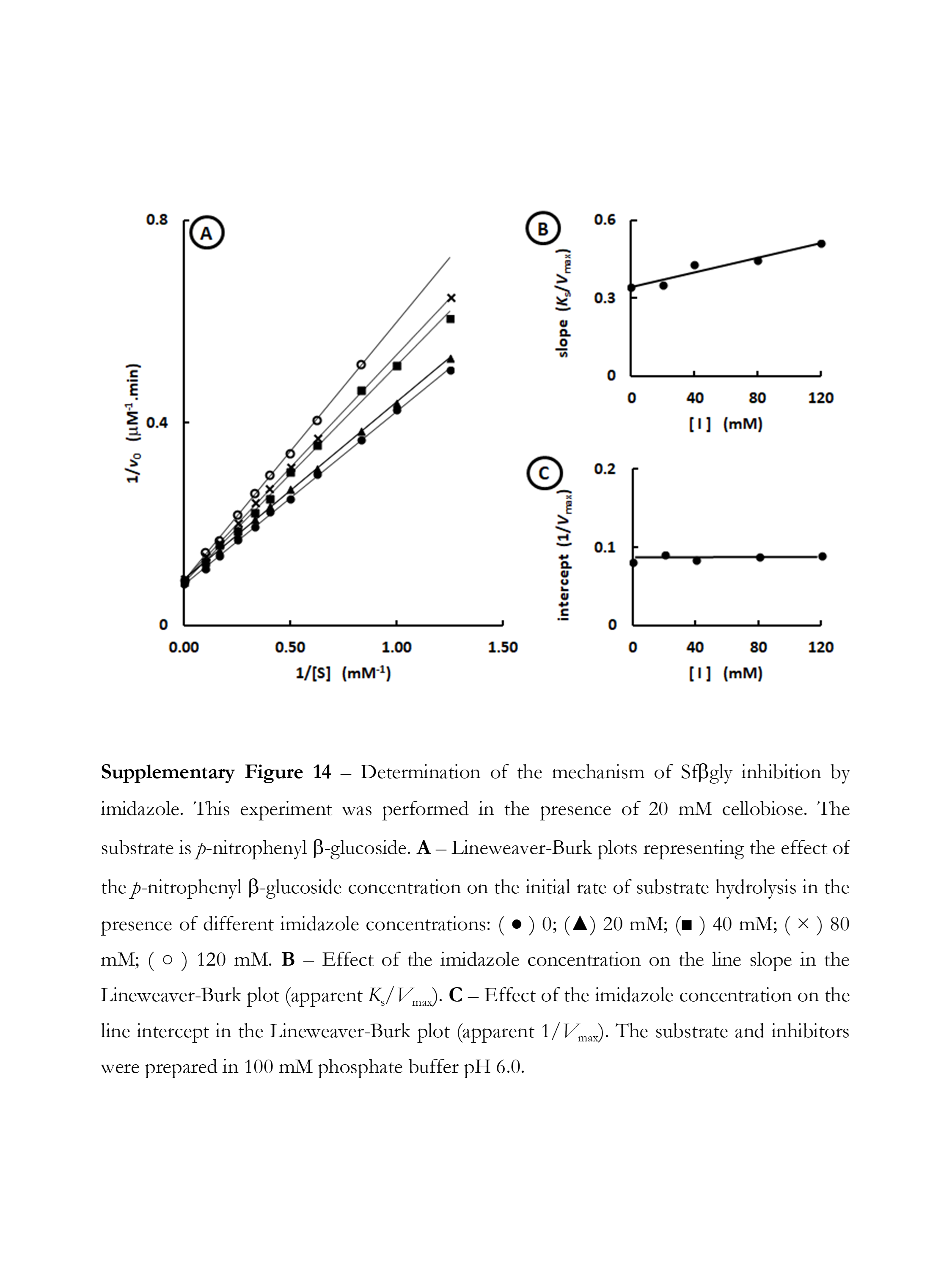

Supplement: Supplementary file 14 — Fig. S14. Determination of the mechanism of Sfβgly inhibition by imidazole in the presence of 20 mM cellobiose. The substrate is p‐nitrophenyl β‐glucoside. [file FEB4-13-912-s016.tif]

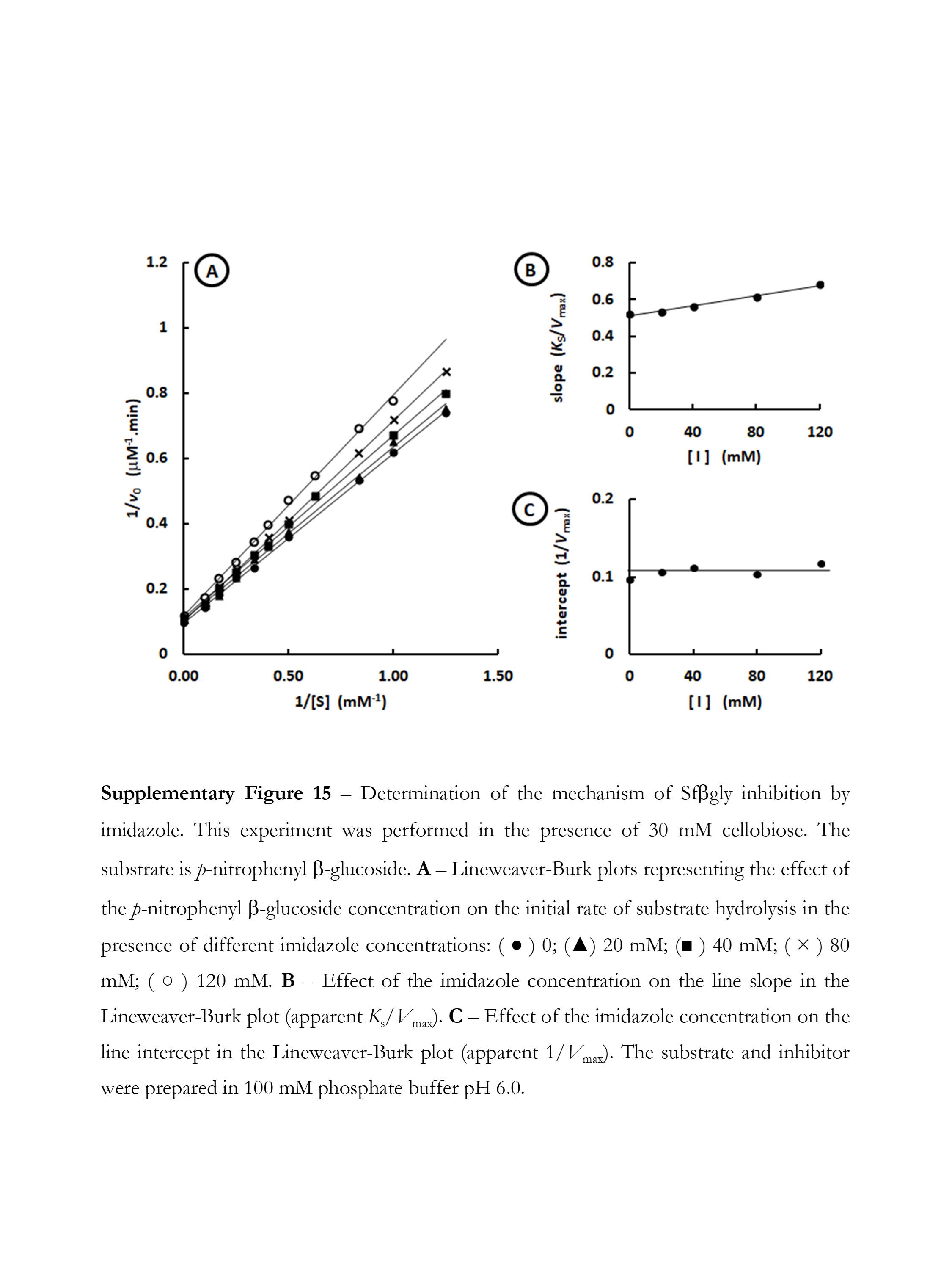

Supplement: Supplementary file 15 — Fig. S15. Determination of the mechanism of Sfβgly inhibition by imidazole in the presence of 30 mM cellobiose. The substrate is p‐nitrophenyl β‐glucoside. [file FEB4-13-912-s017.tif]

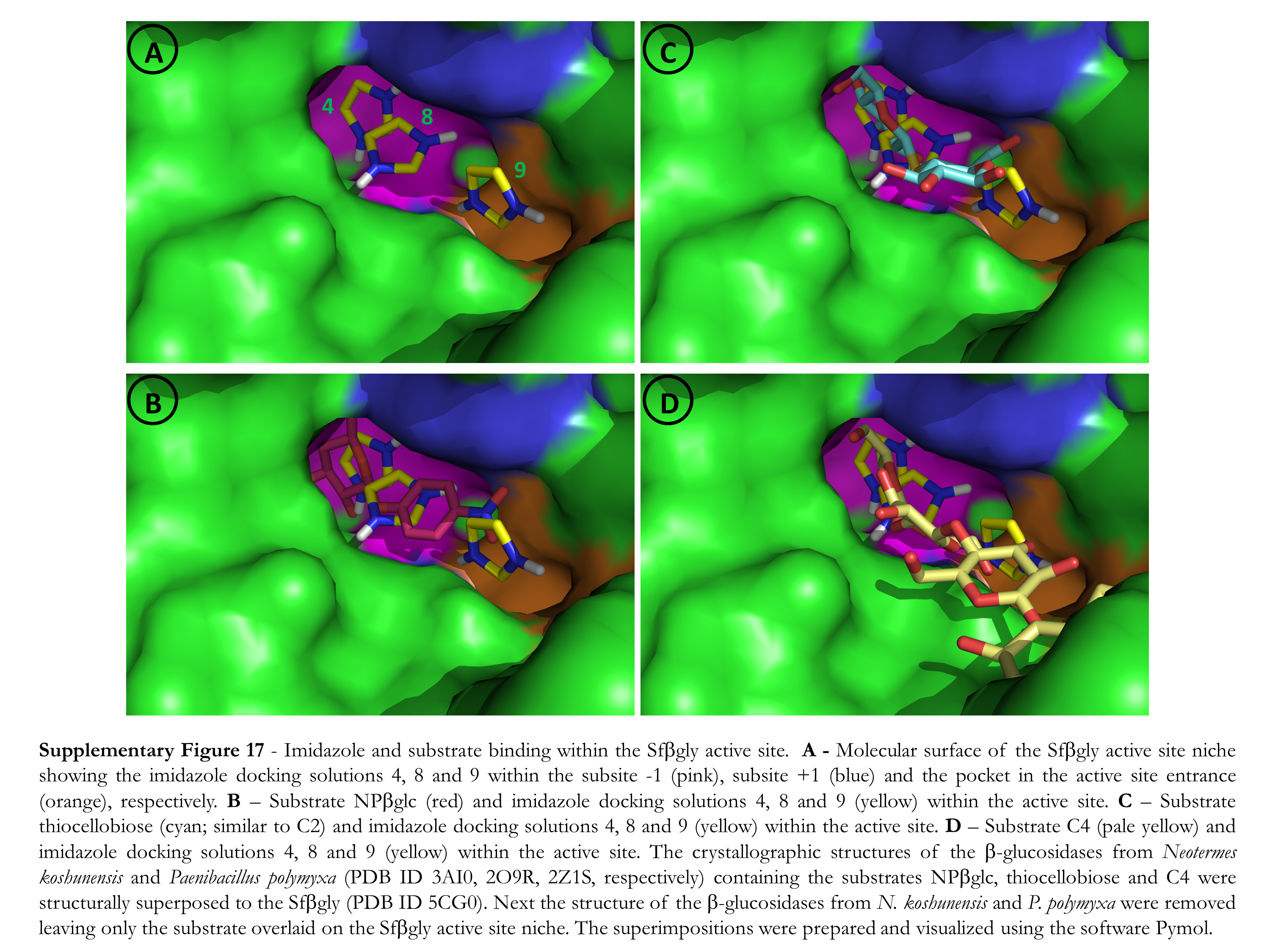

Supplement: Supplementary file 17 — Fig. S17. Imidazole and substrate binding within the Sfβgly active site. [file FEB4-13-912-s014.tif]
