# Supplementary material for: Mechanism of imidazole inhibition of a GH1 β‐glucosidase
Source: FEBS Open Bio. 2023 Mar 25;13(5):912–25. doi: 10.1002/2211-5463.13595 (PMC10153361; doi:10.1002/2211-5463.13595)
Supplement: Supplementary file 16 — Fig. S16. Deduction of the rate equation describing the simultaneous effect of a partial competitive and a competitive inhibitor on the enzymatic hydrolysis of the substrate. [file FEB4-13-912-s013.pdf]

Supplementary Figure 16 – Deduction of the rate equation describing the simultaneous effect of a partial competitive and a competitive inhibitor on the enzymatic hydrolysis of the substrate. I, partial competitive inhibitor (imidazole); J, competitive inhibitor (cellobiose); S, substrate (*p*-nitrophenyl β-glucoside), E, GH1 β-glucosidase Sfβgly; P, product. K_s is the dissociation constant for the enzyme-substrate complex (ES). K_i is dissociation constant for the enzyme-imidazole complex (EI). K_j is dissociation constant for the enzyme-cellobiose complex (EJ). The α factor represents the hindering effect between the substrate and imidazole. The β factor represents the hindering effect between imidazole and cellobiose. If cellobiose and imidazole binding would not present a mutually hindering effect, then β = 1. On the other hand, if cellobiose and imidazole binding would present a mutually hindering effect, then β > 1.

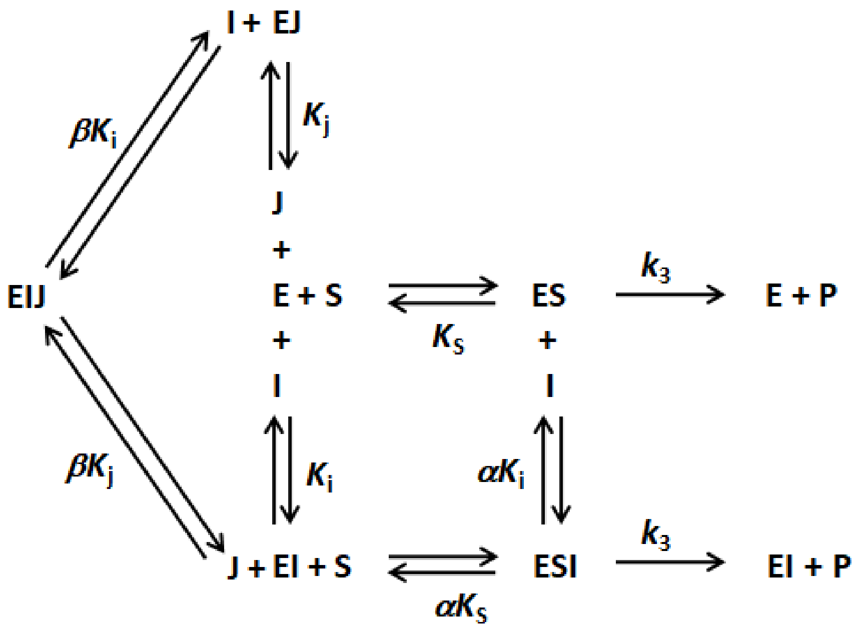

Based on this scheme, the dissociation constants and the respective complexes are defined as seem below.

$$K_S = \frac{[E][S]}{[ES]} \qquad [ES] = \frac{[E][S]}{K_S}$$

$$K_i = \frac{[E][I]}{[EI]} \qquad [EI] = \frac{[E][I]}{K_i}$$

$$K_j = \frac{[E][J]}{[EJ]} \qquad [EJ] = \frac{[E][J]}{K_j}$$

$$\alpha K_S = \frac{[EI][S]}{[ESI]} \qquad [ESI] = \frac{[EI][S]}{\alpha K_S} = \frac{[E][I][S]}{\alpha K_S K_i}$$

$$\beta K_i = \frac{[EJ][I]}{[EIJ]} \qquad [EIJ] = \frac{[EJ][I]}{\beta K_i} = \frac{[E][J][I]}{\beta K_j K_i}$$

$$\beta K_j = \frac{[EI][J]}{[EIJ]}$$

$$\alpha K_i = \frac{[ES][I]}{[ESI]}$$

The initial (v_0) and maximum rate ($V_{\max}$) are expressed as:

$$v_0 \ = \ k_3[ES] + k_3[ESI]$$

$$V_{max} \ = \ k_3[E]_{total}$$

The total enzyme population ($[E]_{total}$) is distributed among the species presented in the scheme above.

$$[E]_{total} = [E] + \ [ES] \ + [ESI] + \ [EI] + [EJ] + \ [EIJ]$$

Based on these definitions the relative rate $v_0/V_{\max}$ is expressed as:

$$\frac{v_0}{V_{max}} = \frac{k_3[ES] + k_3[ESI]}{k_3([E] + \ [ES] \ + [ESI] + \ [EI] + [EJ] + \ [EIJ])}$$

Definitions of the complexes ES, ESI, EI, EJ e EIJ are inserted into the $v_0/V_{\max}$ equation.

$$\frac{v_0}{V_{max}} = \frac{k_3\frac{[E][S]}{K_S} + k_3\frac{[E][I][S]}{\alpha K_S K_i}}{k_3([E] + \frac{[E][S]}{K_S} + \frac{[E][I][S]}{\alpha K_S K_i} + \frac{[E][I]}{K_i} + \frac{[E][J]}{K_j} + \frac{[E][J][I]}{\beta K_j K_i})}$$

The equation above is simplified by eliminating k_3 e [E]

$$\frac{v_0}{V_{max}} = \frac{\frac{[S]}{K_S} + \frac{[I][S]}{\alpha K_S K_i}}{(1 + \frac{[S]}{K_S} + \frac{[I][S]}{\alpha K_S K_i} + \frac{[I]}{K_i} + \frac{[J]}{K_j} + \frac{[J][I]}{\beta K_j K_i})}$$

$$\frac{v_0}{V_{max}} = \frac{\frac{\alpha K_i[S] + [I][S]}{\alpha K_S K_i}}{(1 + \frac{[S]}{K_S} + \frac{[I][S]}{\alpha K_S K_i} + \frac{[I]}{K_i} + \frac{[J]}{K_j} + \frac{[J][I]}{\beta K_j K_i})}$$

$$\frac{v_0}{V_{max}} = \frac{\frac{[S](\alpha K_i + [I])}{\alpha K_S K_i}}{(1 + \frac{[S]}{K_S} + \frac{[I][S]}{\alpha K_S K_i} + \frac{[I]}{K_i} + \frac{[J]}{K_j} + \frac{[J][I]}{\beta K_j K_i})}$$

Numerator and denominator are multiplied by $\alpha K_s K_i$.

$$\frac{v_0}{V_{max}} = \frac{[S](\alpha K_i + [I])}{\alpha K_S K_i + \alpha K_i[S] + [I][S] + \alpha K_S[I] + \alpha K_S K_i \frac{[J]}{K_j} + \frac{\alpha K_S [J][I]}{\beta K_j}}$$

In the denominator, find a common factor in the terms containing K_s e [S].

$$\frac{v_0}{V_{max}} = \frac{[S](\alpha K_i + [I])}{K_S\left(\alpha K_i + \alpha[I] + \alpha K_i \frac{[J]}{K_j} + \frac{\alpha[J][I]}{\beta K_j}\right) + [S](\alpha K_i + [I])}$$

$$\frac{v_0}{V_{max}} = \frac{[S]}{K_S\left(\frac{\alpha K_i + \alpha[I] + \alpha K_i \frac{[J]}{K_j} + \frac{\alpha[J][I]}{\beta K_j}}{\alpha K_i + [I]}\right) + [S]}$$

$$v_0 = \frac{V_{max}[S]}{K_S\left(\frac{\alpha K_i + \alpha[I] + \alpha K_i \frac{[J]}{K_j} + \frac{\alpha[J][I]}{\beta K_j}}{\alpha K_i + [I]}\right) + [S]}$$

The equation above is similar to the Michaelis-Menten equation, hence it is linearized using the Lineweaver-Burk inversion.

$$\frac{1}{v_0} = \frac{K_S}{V_{max}}\left(\frac{\alpha K_i + \alpha[I] + \alpha K_i \frac{[J]}{K_j} + \frac{\alpha[J][I]}{\beta K_j}}{\alpha K_i + [I]}\right)\frac{1}{[S]} + \frac{1}{V_{max}}$$

Considering that only the slope is affected by the inhibitor I and J, the slope is isolated and analyzed in two limiting conditions, [I] = ∞ and [I] = 0.

$$slope = \frac{K_S}{V_{max}}\left(\frac{\alpha K_i + \alpha[I] + \alpha K_i \frac{[J]}{K_j} + \frac{\alpha[J][I]}{\beta K_j}}{\alpha K_i + [I]}\right)$$

If [I] = ∞, only the terms containing [I] are significant to the sum, whereas the others are discarded.

$$slope_{\infty} = \frac{K_S}{V_{max}} \left(\frac{\alpha[I] \; + \; \frac{\alpha[J][I]}{\beta K_j}}{[I]} \right)$$

$$slope_{\infty} = \frac{K_S}{V_{max}} \alpha \left(1 + \frac{[J]}{\beta K_j} \right)$$

If [I] = 0, terms containing [I] are eliminated

$$slope_{0} = \frac{K_S}{V_{max}} \left(\frac{\alpha K_i \; + \alpha K_i \frac{[J]}{K_j}}{\alpha K_i} \right)$$

$$slope_{0} = \frac{K_S}{V_{max}} \left(1 + \frac{[J]}{K_j} \right)$$

Then, the ratio $slope_{\infty}/slope_{0}$ is expressed as:

$$\frac{slope_{\infty}}{slope_{0}} = \frac{\frac{K_S}{V_{max}} \alpha \left(1 + \frac{[J]}{\beta K_j} \right)}{\frac{K_S}{V_{max}} \left(1 + \frac{[J]}{K_j} \right)}$$

$$\frac{slope_{\infty}}{slope_{0}} = \frac{\alpha \left(1 + \frac{[J]}{\beta K_j} \right)}{\left(1 + \frac{[J]}{K_j} \right)}$$

$$\frac{slope_{\infty}}{slope_{0}} = \frac{\frac{\alpha \beta K_j + \alpha [J]}{\beta K_j}}{\frac{K_j + [J]}{K_j}}$$

$$\frac{slope_{\infty}}{slope_{0}} = \frac{\alpha}{\beta} \frac{(\beta K_j + [J])}{(K_j + [J])}$$
