# Supplementary material for: Mechanism of imidazole inhibition of a GH1 β‐glucosidase
Source: FEBS Open Bio. 2023 Mar 25;13(5):912–25. doi: 10.1002/2211-5463.13595 (PMC10153361; doi:10.1002/2211-5463.13595)
Supplement: Supplementary file 18 — Table S1. Kinetic parameters for the hydrolysis of different substrates by Sfβgly. [file FEB4-13-912-s010.docx]

**Supplementary Table 1** – Kinetic parameters for the hydrolysis of different substrates by the Sfβgly

| **Substrate** | ***K*_S_ (mM)** | ***k*_3_ (min^-1^)** | ***k*_3_/*K*_S_ (mM^-1^. min^-1^)** |
| --- | --- | --- | --- |
| NPβglc | 1.73 ± 0.04 | 80 ± 20 | 46 |
| C2 | 4.6 ± 0.6 | 69 ± 15 | 15 |
| C4 | 0.3 ± 0.1 | 90 ± 20 | 362 |

Data are mean and standard deviation based on three independent experiments. (Figures 4 - 6; Supplementary Figures 2 to 10). NPβglc, *p*-nitrophenyl β-glucoside; C2, cellobiose; C4, cellotetraose.
